# Sustainable Immunomodulatory via Macrophage P2Y12 Inhibition Mediated Bioactive Patche for Peritendinous Antiadhesion

**DOI:** 10.1002/advs.202409128

**Published:** 2024-12-04

**Authors:** Zaijin Tao, Shuo Wang, Jingwen Liu, Tonghe Zhu, Jia Jiang, Shen Liu, Xin Ma

**Affiliations:** ^1^ Department of Orthopaedics Shanghai Sixth People's Hospital Affiliated to Shanghai Jiao Tong University School of Medicine 600 Yishan Rd. Shanghai 200233 P. R. China; ^2^ Multidisciplinary Centre for Advanced Materials Institute for Frontier Medical Technology School of Chemistry and Chemical Engineering Shanghai University of Engineering Science 333 Longteng Rd. Shanghai 201620 P. R. China

**Keywords:** foreign body reactions, persistent immunomodulation (PIM), P2Y12, tendon adhesion

## Abstract

Persistent anti‐inflammatory responses are critical for the prevention of peritendinous adhesion. Although modified anti‐adhesion barriers have been studied extensively, the immune response induced by the implants and the unclear mechanism limits their application. In this research, the advantage of the multi‐functionalities of CA (caffeic acid) is taken to synthesize biodegradable poly (ester urethane) urea elastomers with ester‐ and carbamate‐bonded CA (PEUU‐CA). PEUU‐CA is electrospun into bioactive patches that can uniquely present a sustained CA niche, referred to as BPSN. In the early stage of degradation, the breakage of the ester bond from BPSN is the dominant factor contributing to the early release of CA. In the later stage of BPSN degradation, the breakage of the ester and carbamate bonds contributes to the sustained release of CA. In vitro experiments showed that CA, when specifically bound to the P2Y12 receptor, down‐regulated the expression and function of active P2Y12, effectively inhibiting the aberrant activation of macrophages and the secretion of inflammatory chemokines. BPSN addresses the foreign body reaction induced by macrophage‐dominated biomaterial implantation and the issue of the short‐term release of drugs at later stages of adhesion, providing a feasible strategy for the prevention and treatment of tissue adhesion, and more broadly, the well‐known implant‐derived inflammatory responses.

## Graphical abstract

Construction of a long‐lasting anti‐inflammatory bioactive patche with sustainable immunomodulatory using caffeic acid for native functional modification of polyurethane. This structure allows long‐term real‐time modulation of inflammation through the macrophage P2Y12/NF‐κB pathway, leading to effective treatment of tendon adhesion.

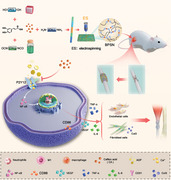

## 1 Introduction

Caffeic acid (CA) is a polyphenol compound that we ingest daily and is widely found in natural plant products.^[^ ^1^, ^2^ ^]^ The sources of CA intake are not limited to those found in coffee, but also in apples, plums, black oranges, or many herbs from the mint subfamily.^[^ ^3^ ^]^ Studies have reported that CA is an essential compound in fighting against various human conditions, such as cancer, diabetes, and atherosclerosis.^[^ ^4^, ^5^, ^6^ ^]^ The presence of a 3‐hydroxyl group and a second hydroxyl in the para position endow CA with excellent anti‐inflammatory and antioxidant properties.^[^ ^7^ ^]^ Akshay et al. reported that micelles containing CA can significantly reduce the severity of inflammatory arthritis.^[^ ^8^ ^]^ Furthermore, CA has been shown to reduce tumor necrosis factor α (TNF‐α), interleukin 6 (IL‐6), cyclooxygenase 2 (COX‐2), and **nuclear factor kappa B (**NF‐κB) levels by inhibiting the expression of peroxisome proliferator‐activated receptors.^[^ ^9^, ^10^ ^]^ P2Y12 is a G protein‐coupled (GPCR) purinoid receptor.^[^ ^11^, ^12^ ^]^ P2Y12 is not only present on the surface of platelets but is also expressed on immune cells such as monocytes,^[^ ^13^, ^14^ ^]^ microglia cells,^[^ ^15^, ^16^ ^]^ tumor‐associated macrophages,^[^ ^17^ ^]^ and hepatic macrophages.^[^ ^18^ ^]^ Inhibition of P2Y12 expression in macrophages has been found to reduce the inflammatory response after cardiac reperfusion, thereby improving myocardial remodeling.^[^ ^19^ ^]^ However, whether P2Y12 is expressed in peritendinous adhesion tissues and P2Y12 plays a role in PAF remains unexplored.

Peritendinous adhesion formation (PAF) is a major clinical complication after tendon operation. The PAF refers to the development of fibrotic tissue between the tendon and peritendinous tissues, which involves scar tissue growing into the injured tendon. As an inflammatory proliferation disease after tendon injury, peritendinous adhesion refers to the overactivity of macrophages in the inflammatory environment.^[^ ^20^, ^21^, ^22^ ^]^ Electrospun fibrous membranes currently constitute the principal therapeutic approach for managing tendon adhesion, with their superiority stemming from their dual role as both a physical barrier against the invasion of adherent tissues and a drug delivery system facilitating sustained release. Furthermore, the optimized pore sizes enable effective nutrient exchange between the membrane and the surrounding environment.^[^ ^23^ ^]^ However, the implantation of biomaterials in the biomaterials degradation process is accompanied by foreign body reaction (FBR).^[^ ^24^, ^25^, ^26^ ^]^ Early stable degradation induces moderate inflammation, accompanied by excessive accumulation of degradation products, leading to severe inflammation at later stages.^[^ ^27^, ^28^ ^]^ Based on this pathological process, the current drug‐loading approach cannot meet the effective regulation of inflammation at the late stage of degradation. This is because the sudden release in the initial stage leads to inefficient or even no release in the later stage.^[^ ^29^, ^30^ ^]^ The drugs have been investigated in previous reports for anti‐adhesion, including nonsteroidal anti‐inflammatory drugs, antimetabolites, and antioxidant stress drugs.^[^ ^31^, ^32^, ^33^, ^34^, ^35^ ^]^ Loading of anti‐inflammatory drugs showed positive inhibitory effects on FBR. Previous studies have attempted to incorporate anti‐inflammatory agents into electrospun fibers through covalent modification or direct blending to achieve sustained release and keep drug activity.^[^ ^36^, ^37^, ^38^ ^]^ Recent ani‐adhesion drugs are very easy to oxidize, resulting in a short half‐life of the active small molecules in the body and, as the release time of these active ingredients is generally within one week, especially, limited drug availability at the later stages of adhesion formation. Additionally, the limited availability of hydroxyl and carboxyl groups in these drugs restricts chemical bonding, leading to inadequate drug‐loading capacity and an inability to fulfill persistent anti‐adhesion requirements. Therefore, a long‐term effective anti‐inflammatory ecosite is essential to prevent FBR and adhesion formation, especially at a later stage. This implies the need to design novel biomaterials capable of releasing drugs along with material degradation to alleviate the abnormal immune microenvironment.

In this research, we perform native functional modification of degradable polyurethanes to synthesize a bioactive patch for sustained immunomodulation by using CA drug molecules as soft segment portions. Biodegradable elastomers named PEUU‐CA were synthesized using poly(Ɛ‐caprolactone) diol (PCL diol, HO‐PCL‐OH), caffeic acid (CA), hexamethylene diisocyanate (HDI) as soft segments and 1, 4‐butanediamine (BDA) as chain extender. These elastomers were then electrospun into bioactive patches featuring a sustainable CA niche, referred to as BPSN. CA is locally released from the biopatch to modulate macrophage infiltration until the material is completely degraded. In the early release phase in vitro, the CA‐linked ester bond degradation acts as an early responsive dominant for CA release to control inflammation, while in the later phase, the breakage of ester and carbamate bonds both contribute to the release of CA for the control of severe FBR and recurrence of peritendinous adhesion. In addition, we applied CA for the first time in the prevention and treatment of tendon adhesion and revealed for the first time the interaction between CA and the P2Y12 receptor. In vitro experiments showed that CA, when specifically bound to P2Y12 receptor, down‐regulated the expression and function of active P2Y12. Inhibition of macrophage Ca^2+^ overload‐induced inflammatory responses via the P2Y12/NF‐κB signaling pathway. Comprehensive studies were performed to investigate the sustained anti‐inflammatory effects and the inhibition of endothelial cell activation of the BPSN treatment. Additionally, the biotherapeutic effects of BPSN were investigated in a rat Achilles tendon injury model to demonstrate the compelling performance for peritendinous anti‐adhesion.

## 2 Results

### 2.1 P2Y12 Expression on the Surface of Macrophages and Molecular Docking Simulations

**Figure 1:**
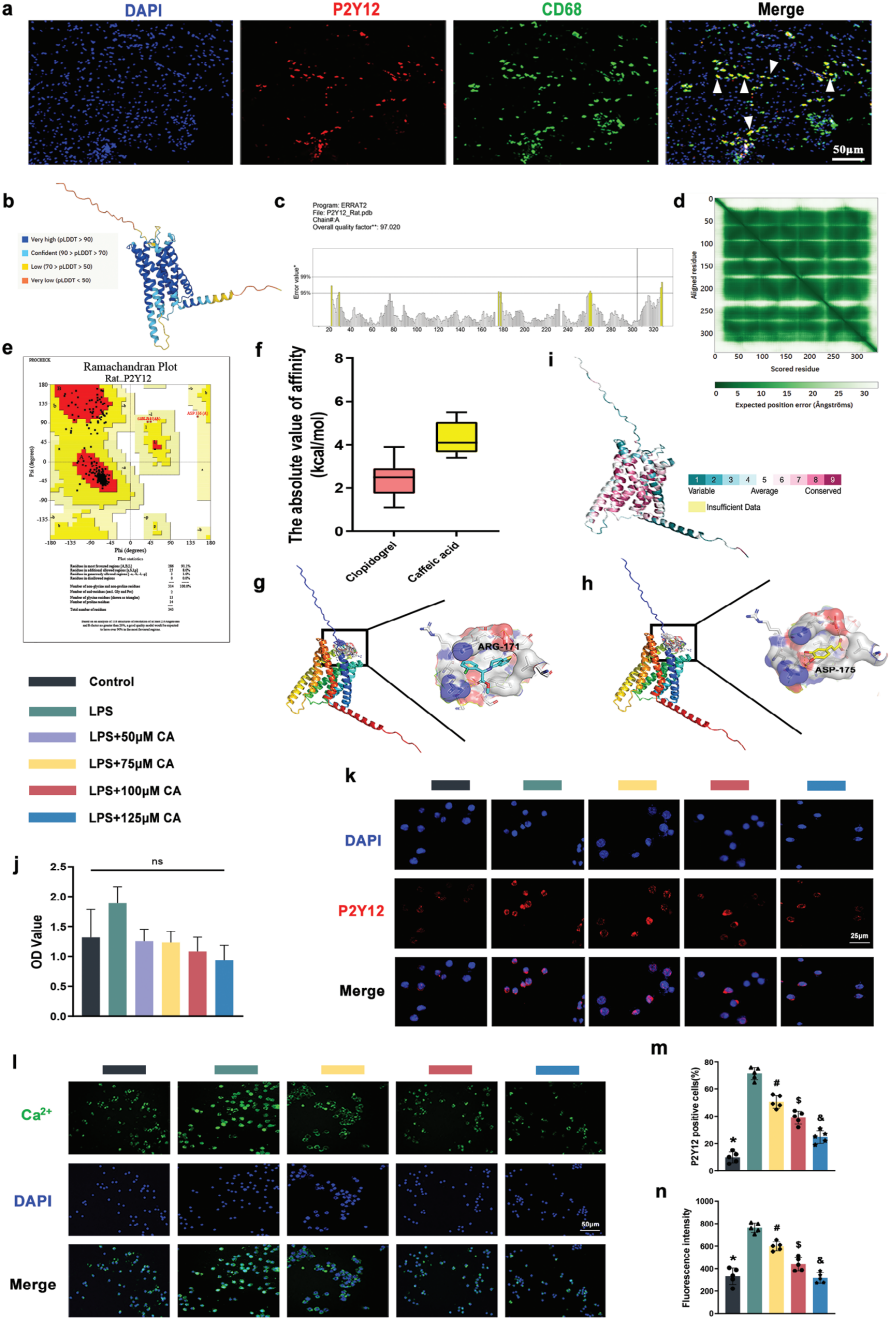
The distribution of P2Y12 in vivo and P2Y12 molecular docking with CA and clopidogrel. a) in vivo verification, fluorescent double‐labeling staining of CD68/P2Y12 in 21‐day rat Achilles tendon adherent tissues. b) The crystal structure and LDDT scores of P2Y12. c) ERRAT was used to evaluate the patterns of nonbonded atomic interactions of P2Y12. d) The structure rationality of amino acid residues was assessed by a Predicted aligned error plot and e) Ramachandran plot. f) The affinity scores of CA or clopidogrel to P2Y12. g) 3D diagram of the docking pose of clopidogrel to P2Y12 was shown. h) The best‐docked conformation between CA and P2Y12. CA binds toward P2Y12 by forming hydrogen bonds with ASP‐175 residues. i) Conservation analysis of amino acid proteins. j) Macrophage activity was assayed under the treatment of different concentrations of CA (50, 75, 100, 125 µM). The CA concentrations of 75, 100, and 125 µM were selected for subsequent experiments. k) DAPI/P2Y12 fluorescence staining images. l) Fluorescence images of labeled Ca^2+^/DAPI using calcium ion fluorescent probe. m) Statistical quantitative analysis of the P2Y12‐positive cells in each group. n) Fluorescence intensity statistics of labeled calcium ion endocytosis in each group. (Data are representatives of independent experiments and all data are given as means ± SD; *n* = 5 per group (k‐n); ns: non‐significant, *, #, $, & *p* < 0.05 compared to the LPS group).

An analysis of human mRNA levels and proteomic findings indicates that P2Y12 receptors are widely distributed in various tissues and organs throughout the human body (Figure S1A–E, Supporting Information). Notably, a compelling discovery emerged, revealing a concentration of P2Y12 receptors on immune cells, including microglia cells, Kupffer cells, and macrophages (Figure S1D, Supporting Information). In light of this observation, we speculated whether the activation of P2Y12 receptors in macrophages is linked to the formation of adherent tissue. We constructed a rat Achilles tendon injury model, and after 21 d, we collected the material and conducted CD68/P2Y12 double‐labeled fluorescence staining (**Figure** **1**a). Additionally, At the cellular level, we mimicked the expression of P2Y12 receptors on the surface of macrophages in an inflammatory environment in vivo through LPS stimulation. As depicted in (Figure S2, Supporting Information), we observed a significant up‐regulation of P2Y12 expression in the LPS group compared to the control group. Our findings revealed a high expression of P2Y12 in the adherent tissue. From this, we postulated that macrophage P2Y12 receptors are activated in the inflammatory environment, contributing to the formation of adherent tissues during the repair of injured tissues.

Previous studies have suggested that the function and expression of P2Y12 may represent a crucial target for the release of proinflammatory factors associated with M1 macrophage polarization.^[^ ^39^ ^]^ To investigate the interaction between CA and P2Y12 receptors in the initiation of inflammation and the formation of peritendinous adhesion tissues, we conducted AI‐based modeling of the P2Y12 protein structure and molecular docking of CA with P2Y12. The Alphafold v2.0 database predicted the optimal protein crystal structure of P2Y12, meeting the criteria of characteristic atomic interactions and probability density function energy requirements detected by ERRAT and Ramachandran plots (Figure 1b–e). In addition, the Local Distance Difference Test (LDDT) score and predicted alignment error maps indicated that the major residues of P2Y12 were distributed in regions greater than 50 pLDDT and in the dark green region, signifying low error. (Figure 1f–h) displays the affinity scores and binding positions. The absolute affinity scores of CA with P2Y12 franged from 3.4 to 5.5 kcal mol^−1^, while those of clopidogrel (a specific inhibitor of P2Y12) were 1.1‐3.9 kcal mol^−1^ (*P* < 0.001). CA exhibited higher affinity. The simulated docking results demonstrated a perfect match between CA and P2Y12, allowing CA to bind specifically to P2Y12 residues by forming hydrogen bonds on amino acid residue ASP‐175. The results of the conservation analysis of amino acid proteins indicated a high degree of applicability of our findings across species (Figure 1i).

The effects of caffeic acid (CA) on the in vitro toxic effects and viability of macrophages are illustrated in (Figure 1j). RAW264.7 macrophages were co‐incubated with CA for 24 h at concentrations of 50, 75, 100, and 125 µM. The OD values of the LPS group served as a control. No significant difference in cell viability was observed between the control, LPS, and 50–125 µM CA concentration groups. Based on our findings and the literature, we selected CA concentrations of 75 µM, 100 µM, and 125 µM for subsequent experiments.^[^ ^40^ ^]^ We investigated whether the expression of the P2Y12 receptor was associated with macrophage activation. (Figure 1k,m) demonstrate that, under the same treatment conditions, the expression of P2Y12 was accompanied by a dose‐dependent CA. P2Y12, acting as a ligand‐gated channel, triggered an increase in calcium mobilization upon activation by ADP.

To assess whether the function of P2Y12 was affected, we measured the inward flow of intracellular calcium ions using the Calcium Ion Probe Kit, with green fluorescent labeling for Ca^2+^ and blue DAPI labeling for the nucleus. (Figure 1l) shows a decrease in the intensity of Ca^2+^ fluorescent labeling after CA addition. Quantifying the green fluorescence intensity (Figure 1n) revealed that CA significantly inhibited extracellular Ca^2+^ influx. As an important mediator of intercellular signaling, Ca^2+^ plays a role in regulating various biological responses in cells. Our results indicate that CA intervention inhibited the activation and expression of P2Y12, and calcium mobilization was significantly reduced. This in vitro evidence supports the notion that CA can effectively replace clopidogrel, binding specifically to P2Y12 for therapeutic purposes.

### 2.2 Macrophage Polarization Mediated by P2Y12 Activation In Vitro

**Figure 2:**
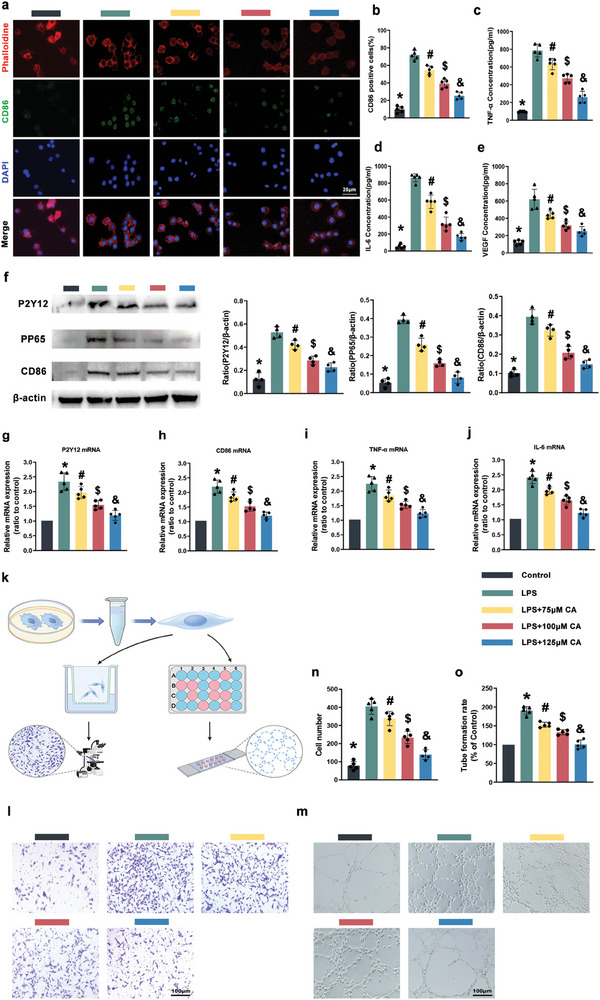
The toxic effects of different concentrations of CA on macrophages, and in vitro anti‐inflammatory properties were verified. Crosstalk between treated macrophages and endothelial cells. The LPS group was simulated in vivo inflammatory microenvironment. a,b) CD86/Phalloidine fluorescence double staining was used to analyze the anti‐inflammatory performance of CA at different concentrations, and the positive cellular CD86 expression rate statistics. c–e) Cell supernatants were extracted, and the expression of TNF‐α, IL‐6, and VEGF was detected in the cell supernatants of each group by using Elisa kit. f) Western blot analysis showed that inhibition of P2Y12 was accompanied by a decrease in the phosphorylation level of NF‐κB. g–j) Real‐time PCR results showed that mRNA levels of relevant inflammatory indicators were significantly higher in the LPS group and decreased in the CA‐treated group. k) Experimental design to study macrophage association with endothelial cells. l) Crystalline violet‐stained image of an endothelial cell passing through a small compartment. m) Image of endothelial cell tube formation in Matrigel. n) Quantitative statistics of cell migration number. o) Analytical statistics of the rate of cell tube formation. (*, #, $, & *p* < 0.05 compared to the control). (Data are representatives of independent experiments and all data are given as means ± SD; *n* = 5 per group (b–o), *n* = 5 (f); *, #, $, & *p* < 0.05 compared to the LPS group).

We evaluated macrophage polarization mediated by P2Y12 and inflammatory cytokine release. Macrophage polarization was assessed by immunofluorescence staining. As depicted in (**Figure** **2**a,b), the classical polarization of macrophages (increased CD86 expression) induced by LPS was observed, and the percentage of CD86 positive cells gradually decreased with increasing CA concentration. This suggested that, under the same treatment conditions, CA suppressed the activation of P2Y12 and subsequent CD86 expression.

Following successful macrophage polarization, cells were co‐incubated with CA for 24 h, and the supernatants were collected to determine the concentrations of TNF‐α, IL‐6, and VEGF in each subgroup by ELISA (Figure 2c‐e). Concentrations of TNF‐α, IL‐6, and VEGF in the LPS group were significantly up‐regulated, surpassing those in the control group. However, with the addition of CA, the concentrations of TNF‐α, IL‐6, and VEGF exhibited a gradient decrease. In addition, mRNA (Figure 2g‐j) and protein expression (Figure 2f) assays revealed that CA largely inhibited M1 polarization by suppressing the activation of P2Y12, thereby reducing the phosphorylation level of NF‐κB. Given the persistence of M1 macrophage and the principles of macrophage polarization, these results imply that CA can dose‐dependently inhibit the expression of the macrophage M1 phenotypic marker CD86 and the release of related inflammatory and growth factors, confirming its anti‐inflammatory and anti‐vascularization effect.

The above conclusions are the result of a study using the RAW264.7 macrophage cell line. To demonstrate the generalizability of CA, we selected the mouse‐derived Bone Marrow‐Derived Macrophage (BMDM) which more closely resembles the physiological structure of the species for validation and analysis. The results were consistent as expected, the positive rate of P2Y12 receptor expression on the surface of BMDM decreased as the concentration of CA action increased (Figure S3, Supporting Information). In addition, we have continued to detect iNOS, another marker of M1 macrophage. As shown in (Figure S4, Supporting Information), the number of iNOS‐positive cells was the highest in the LPS group, while exhibiting a concentration‐dependent reduction with the addition of CA.

### 2.3 Treated Macrophages Regulate Endothelial Cell Migration and Tube Formation

As the inflammatory phase progresses, the activation of inflammatory vascular endothelial cells becomes pivotal in the tissue repair phase. We utilized the supernatant of CA‐treated macrophages and co‐cultured them with human umbilical vein endothelial cells (HUVECs) to assess their migration and tube‐forming ability, respectively (Figure 2k).

In (Figure 2l,n), after endothelial cells were co‐cultured with supernatant in Transwell chambers for 24 h, the number of cell migration was observed using crystal violet staining labeling. The number of cells in the LPS group was significantly higher than in the control group, while the number of cells in the drug‐treated group was significantly lower than in the LPS group.

In tube formation assay (Figure 2m,o), endothelial cells (ECs) co‐incubated with supernatant for 24 h were planted in Matrigel to observe the number of cellular vascularization. The number of ECs vascularized in the LPS group was higher, while the vascularization ability of the experimental group was significantly weakened. These results suggest that CA may reduce the activity of ECs by inhibiting the release of relevant inflammatory factors or VEGF from M1, thereby attenuating the stimulation of ECs. However, the specific mechanism remains unclear.

### 2.4 Characterization of Nanofiber Patches

**Figure 3:**
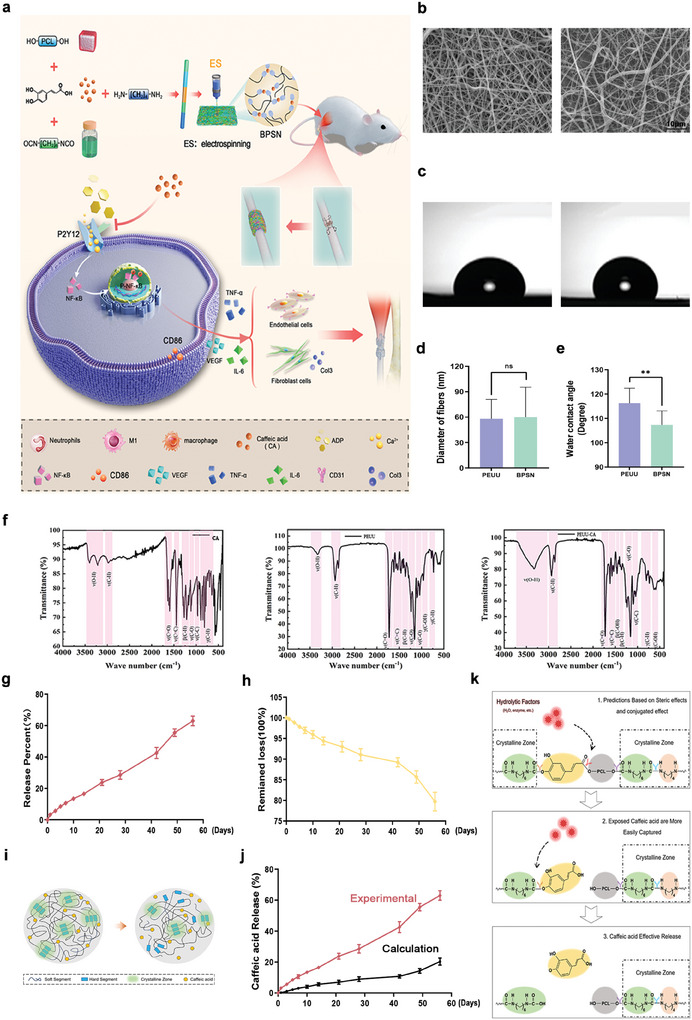
Performance characterization of PEUU and BPSN, degradation of BPSN, and release of CA. a) Schematic diagram of the synthesis and the specific mechanism of action of BPSN. b) Microstructure and d) nanofiber diameter statistics of PEUU and BPSN. c) Hydrophilic/hydrophobic properties and e) water contact angle of PEUU and BPSN. f) FTIR spectra of CA, PEUU, and PEUU‐CA (BPSN). g) Cumulative release rate profiles of the BPSN patch CA. h) Weight‐loss curve of BPSN degradation. i) Schematic of the intrinsic mechanism of CA release in BPSN. j) Comparison of actual and theoretical CA release rates in BPSN. k) Schematic of the mechanism of CA release in BPSN. (Data are representatives of independent experiments and all data are given as means ± SD; *n* = 5 per group; ns: non‐significant, *p* > 0.05; *p < 0.05; **p < 0.01; ***p < 0.001).

Our previous results demonstrated that CA effectively reduces the inflammation response by inhibiting macrophage and endothelial activation. (**Figure** **3**a) describes the synthesis of BPSN in this study and the specific mechanism of CA action. To achieve persistent release of CA, PEUU, and PEUU‐CA (BPSN) were prepared by electrospinning and were subjected to infrared spectroscopy analysis, revealing that the stretching vibration of OH at 3335.39 cm^−1^ in the PEUU‐CA (BPSN) infrared spectrum exhibited increased intensity and width. This suggested an elevated hydroxyl group content in the structure, possibly originating from CA (Figure 3f).

The surface morphology and average size of PEUU and PEUU‐CA (BPSN) were determined by SEM (Figure 3b). The average fiber diameter of PEUU was 58.26 ± 20.39 nm, and for BPSN, it was 60.74 ± 31.62 nm (Figure 3d). These results indicated that CA had good solubility and was stable in the spinning solution system. The water contact angles of PEUU and BPSN were 116.33 ± 4.96^°^, and 107.33± 4.72^°^, respectively (Figure 3c,e). The porosities of PEUU and BPSN were 74.22% and 70.41%, respectively. Mechanical property evaluation indicated that the grafting of CA did not significantly affect the diameter, porosity, water contact angle, and tensile strength of PEUU and BPSN. Tensile strain‐displacement graphs showed similar maximum bearing loads for both materials (Figure S7, Supporting Information).

The release of CA and the weight loss curves of the BPSN during degradation are shown in (Figure 3g,h). The drug release rate remains stable, and with the degradation of BPSN, the cumulative drug release after 8 weeks was 63.89 ± 2.56%, with a BPSN weight loss was 20.29 ± 1.84%. In addition, our release curves show an accelerated release rate after 42 d. (Figure 3i) was a schematic of the intrinsic mechanism of CA release in BPSN. As shown in (Figure 3j), it is found that the actual CA release is higher than the theoretical value. This situation may be due to more degradation reactions at the CA site, which is mostly in the amorphous region of the polyurethane molecular chain, which is more prone to degradation reactions than the molecular chain in the crystalline region. Therefore, the amount of CA released during the previous degradation‐release process is higher than that calculated theoretically. As a result of the existence of the conjugate effect and steric effect, caused the ester bond rupture priority (Figure 3k). As the molecular chain collapses, the carbamate bond starts to break. In addition, based on the schematic diagram of the synthesis and degradation of BPSN, it was shown that the breakage of the ester bond is the dominant factor for the slow release of CA in the early stage, while the ester and carbamate bonds both dominate the late stage of BPSN degradation (Figures S5‐S6, Supporting Information). Therefore, the novel CA‐grafted nanofiber patches (BPSN) could facilitate sustained and slow drug release into surrounding tissues after in vivo implantation.

### 2.5 Effect of In Vitro Drug Release on Macrophage Polarization and Inflammatory Factor Release

**Figure 4:**
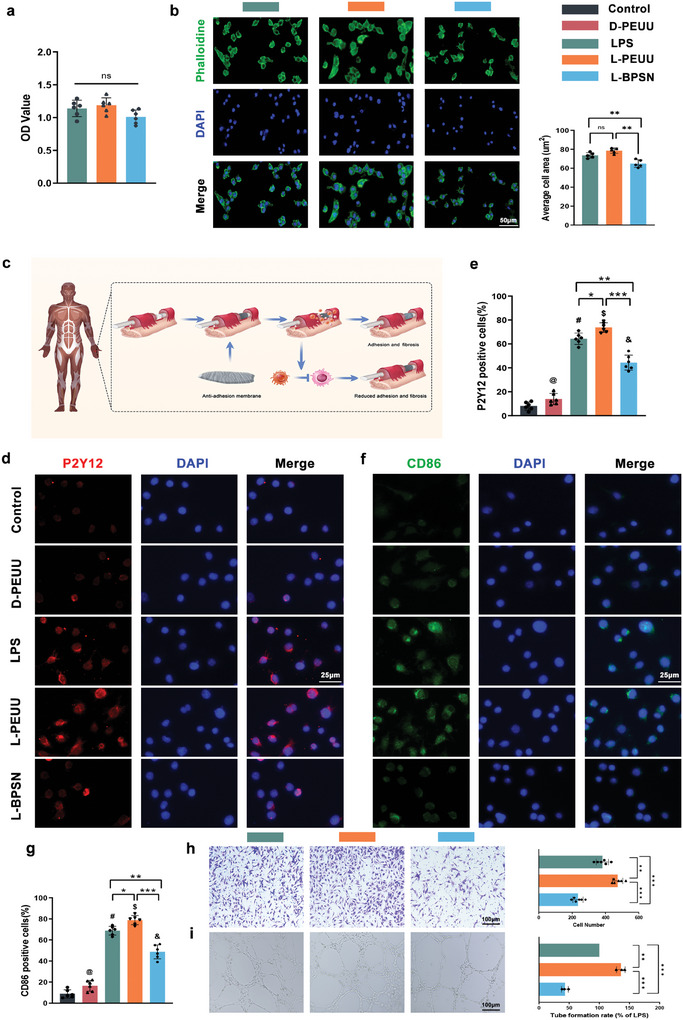
In vitro study of PEUU/BPSN degradation. Macrophages were treated by taking PEUU/BPSN degradation solution. a) Proliferative activity of macrophages. b) Adhesion effects of PEUU and BPSN degradation solution on macrophages and statistics of the adhesion area of the macrophages (*n* = 5). c) Graft‐induced recruitment of immune cells leads to fibrotic encapsulation of tissue interface‐grafts; phenotypic modulation targeting M1 macrophages reduces adhesion and fibrosis. d) P2Y12/DAPI fluorescence staining image. e) Quantitative statistics of P2Y12‐positive cells. f) CD86/DAPI fluorescence staining images. (D‐PEUU represents PEUU degradation products; L‐PEUU and L‐BPSN represent LPS and PEUU or BPSN degradation products respectively.) g) Quantitative statistics of CD86‐positive cells. h) Observation and quantitative analysis of endothelial cell migration crystal violet staining. i) Endothelial cell tube formation images and quantitative analysis (*n* = 3). (@, #, $, & *p* < 0.05 compared to the control). (Data are representatives of independent experiments and all data are given as means ± SD; *n* = 6 per group; ns: non‐significant, *p* > 0.05; *p < 0.05; **p < 0.01; ***p < 0.001).

The published literature indicates that the in vivo application of PEUU leads to material degradation, causing changes in the immune microenvironment, and the resulting degradation monomers can promote macrophage polarization.^[^ ^41^, ^42^ ^]^ (**Figure** **4**c) shows that implant‐induced foreign body reaction exacerbates tissue adhesion and fibrosis. Inhibition of M1 overactivity attenuates adhesion and fibrosis. To assess the drug release effects of the materials, we employed the leachant of PEUU and BPSN after 6 weeks of release to treat macrophages, observing changes in the biological responses of these cells.

First, we detected the effect of the extracts on the proliferative viability of the cells, as shown in (Figure 4a), and found no significant difference between the groups. And the adhesion of macrophages in each group was observed. As illustrated in (Figure 4b), macrophages in the control and L‐PEUU groups exhibited well‐organized cytoskeletal arrangement and expandability, while the cell area was significantly reduced in the L‐BPSN group. Quantitative analysis of the adhesion area in each group revealed that the cell spreading area of the L‐PEUU group was higher than that of the control group, although not significantly different. However, the cell spreading area of the L‐BPSN group was significantly lower than that of the first two groups. Next, we conducted immunofluorescence staining of the cells in each group (Figure 4d,f). We observed that the degradation products of PEUU exhibited some pro‐inflammatory potential compared to the control group, and the addition of PEUU degradation products further promoted the expression and secretion of inflammatory genes after LPS stimulation. The results revealed that the number of positive cells for CD86 and P2Y12 was significantly higher in macrophages treated with PEUU extract compared with the LPS group, while the L‐BPSN group showed the opposite trend (Figure 4e,g). Changes in the concentration of TNF‐α and IL‐6 in the supernatant were detected using the Elisa assay (Figure S10, Supporting Information). As expected, inflammatory factors were increased in the L‐PEUU group, and their expression was significantly reduced in the L‐BPSN group.

Continuing the incubation for 24 h after changing the solution, we extracted the supernatant to co‐culture with endothelial cells (ECs). As the results in (Figure 4h,i) demonstrated that the cell migration number and angiogenesis number were up‐regulated in the L‐PEUU group compared with the LPS group, while the exact opposite was observed in the L‐BPSN group.

Taken together, these experimental results indicate that the degradation of PEUU further induces macrophage activation and promotes the expression of relevant inflammatory cytokines. The secreted inflammatory chemokines might be related to the activation of ECs, stimulating the migration and vasculogenic ability of ECs. On the other hand, the application of BPSN alleviates the excessive development of inflammation, suggesting that CA could be continuously and stably released from the BPSN, exerting its anti‐inflammatory effects and mitigating the adverse consequences of persistent or excessive inflammation.

### 2.6 In Vivo Animal Studies

**Figure 5:**
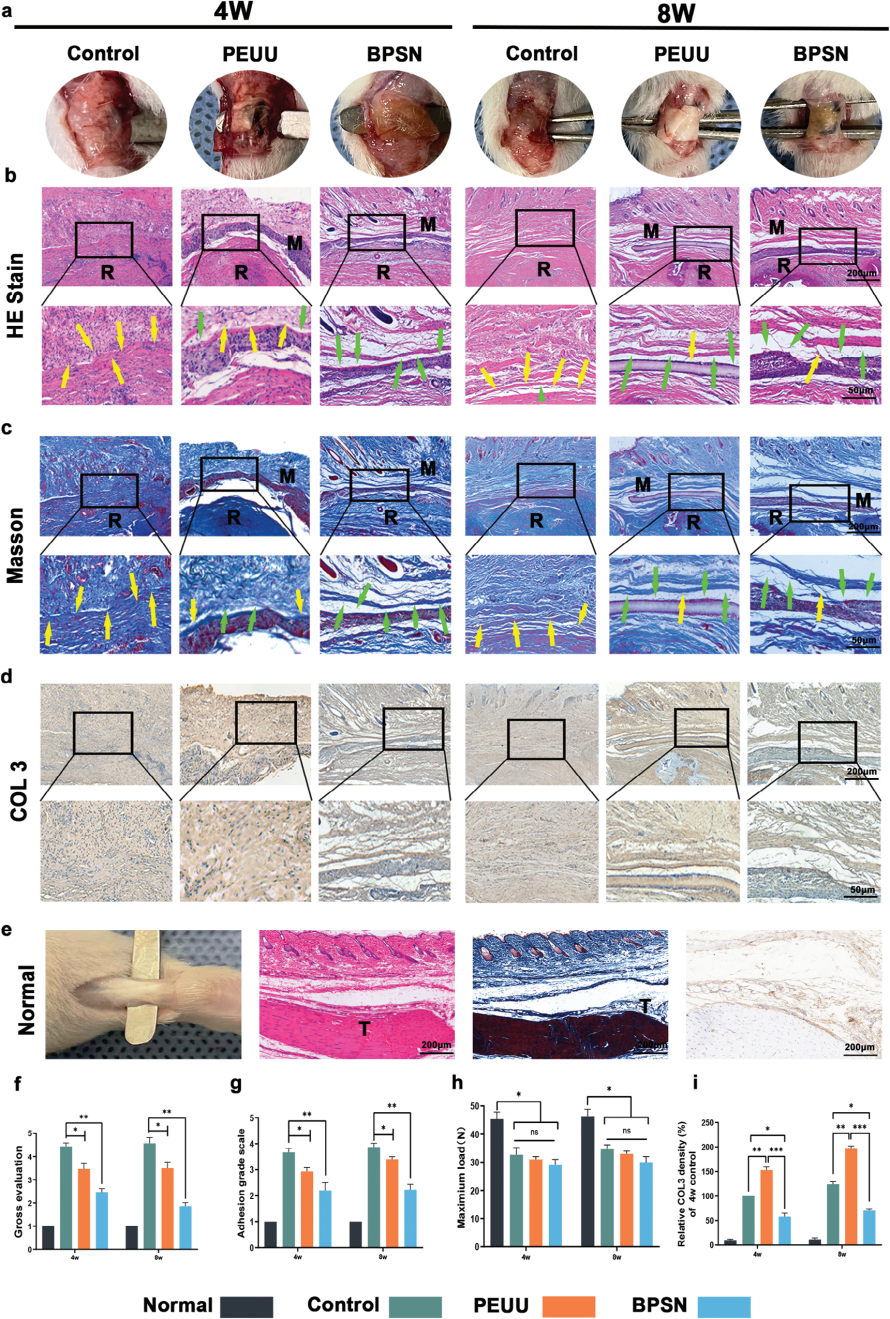
Implantation of BPSN improved the anti‐adhesive efficacy of Achilles tendon injury in rats. a) General image of the rat Achilles tendon injury group, PEUU, BPSN implantation group, 4 w and 8 w after treatment. b) HE staining of rat Achilles tendon adhesion at 4 w and 8 w. c) Representative Masson's trichrome staining plots at 4 w and 8 w after surgery. Yellow arrows indicate adhesion areas, and green arrows indicate areas without adhesion. R: repaired site, M: residual materials. T: tendon. d) Immunohistochemical staining of 4 w tissues and 8 w. e) General image of the rat Achilles tendon normal group, and HE, Masson staining and immunohistochemical staining for COL 3. f) Total score of adhesive tissue around the rat Achilles tendon. g) Histomorphological score of peritendinous adhesion. h) Evaluation of the maximum mechanical load of the regenerated tendon. i) Quantitative analysis of COL3 deposition in peritendinous tissues. (Data are representatives of independent experiments and all data are given as means ± SD; *n* = 3 per group; ns: non‐significant, *p* > 0.05; *p < 0.05; **p < 0.01; ***p < 0.001).

To assess the actual impact and effectiveness of PEUU and BPSN in vivo for peritendinous anti‐adhesion, we wrapped both PEUU and BPSN around the ruptured Achilles tendon of rats and conducted histopathological analyses at 4 w and 8 w postoperative time points. At both 4 w and 8 w postoperatively, we observed a substantial formation of dense adherent tissue around the repaired tendon (**Figure** **5**b,c). This adherent tissue formation was evident even under visual observation (Figure 5a). In contrast, the tissue around the repaired area wrapped with PEUU or BPSN was significantly less dense, allowing for easy blunt separation. The total adhesion scores for each group confirmed these observations (Figure 5f,g). Furthermore, we observed no massive cellular infiltration and significant collagen deposition around the normal rat Achilles tendon (Figure 5e). The maximum tendon carrying load of each group at 4 w and 8 w was depicted in (Figure 5h). Additionally, the maximum tensile strength of the tendons did not significantly differ between the groups at either 4 w or 8 w. Although all of them are lower than the normal tendon group, our intention was not to promote tendon healing, but to improve peritendinous anti‐adhesion. Nonetheless, the result indicated that the implantation of PEUU or BPSN had no side effect on tendon healing.

Although the use of PEUU reduced the degree of peritendinous adhesion, immunohistochemical results revealed a significant increase in collagen deposition around the peritendinous. As shown in (Figure 5d,i), the mean density of COLIII in the peritendinous tissue was analyzed semi‐quantitatively using immunohistochemical staining. In comparison with the control group, the adhesion indicator COLIII increased at all time points due to the application of the PEUU membrane. The BPSN group, however, reversed the pathological changes induced by the PEUU membrane, confirming the in vivo role of CA in decreasing peritendinous COLIII deposition and improving anti‐adhesion efficacy.

**Figure 6:**
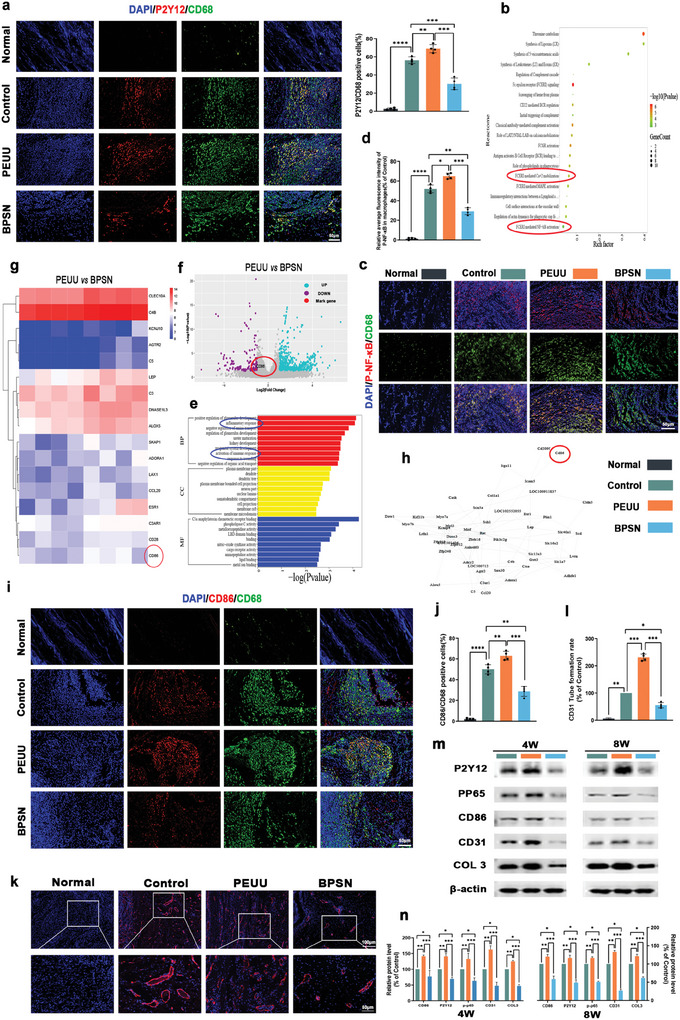
Results of RNA‐seq and implantation of BPSN improves the environment of persistent inflammatory infiltration in a rat Achilles tendon injury model. a) CD68/P2Y12 immunofluorescence double staining results at 8 w postoperatively and quantitative statistics of CD68/P2Y12 positive cell rate. b) Significantly enriched Reactome scatter plots indicate down‐regulation of calcium mobilization and NF‐κB phosphorylation. c, d) Immunofluorescence double staining results of CD68/p‐NF‐κB and quantitative analysis at 8 w postoperatively. e) Functional analysis of GO in the target gene set and significantly enriched GO histograms showed reduced activation of both inflammatory and immune responses. f‐h) Volcano maps for PEUU group and BPSN group related gene screening, heat maps, and target gene protein interaction network diagram. i) Immunofluorescence double staining results of CD68/CD86 at 8 w postoperatively. j) Quantitative statistics of CD68/CD86 positive cell rate. k) CD31/DAPI immunofluorescence staining results 8 w after surgery. l) Quantification of CD31 expression. m, n) Detection and quantitative analysis of CD86, P2Y12, P‐P65, CD31, and COLIII protein expression in peritendinous tissues at 4 w and 8 w after surgery. (Data are representatives of independent experiments and all data are given as means ± SD; *n* = 4 per group; ns: non‐significant, *p* > 0.05; *p < 0.05; **p < 0.01; ***p < 0.001; ****p < 0.0001).

Furthermore, we conducted a whole transcriptome analysis through RNA sequencing (RNA‐Seq) using adherent tissues of PEUU and BPSN from an 8‐week rat Achilles tendon injury model. As depicted in (**Figure** **6**b), significantly enriched reactome scatter plots indicate down‐regulation of calcium mobilization and NF‐κB phosphorylation in BPSN group. (Figure 6e) indicated that inflammatory and immune activation responses were the most affected biological processes in the BPSN group compared to the PEUU group. RNA‐seq results indicate that CA can be effectively released in BPSN to down‐regulate calcium ion mobilization and reduce the phosphorylation level of NF‐κB, thus inhibiting the excessive inflammation or immune response. This included a more pronounced down‐regulation of inflammatory factors such as CD86 (Figure 6f–h).

We further analyzed the activation status of macrophages in sustained inflammatory responses or foreign body responses triggered by PEUU implantation degradation and the regulatory mechanism of BPSN implantation interfering with P2Y12/NF‐κB phosphorylation‐mediated macrophage polarization. As shown in (Figure 6a,c,I,k), we performed immunofluorescence staining on 8‐week tissue sections for double‐labeled P2Y12, p‐NF‐κB, CD86, and single‐labeled CD31. Consistent with the in vitro experiments, there was still a large number of macrophages infiltrating around the tendon wrapped with PEUU 8 w after the repair of the injured tendon. With activation of the P2Y12 receptor, NF‐κB phosphorylation into the nucleus and mediates M1 polarization of macrophages (Figure 6c,d). There was a significant increase in the number of P2Y12 and CD86‐positive macrophages around the peritendinous area in the PEUU group compared with the control group (Figure 6a,j). Additionally, the expression of the angiogenesis marker CD31 was correspondingly up‐regulated in the PEUU group (Figure 6l). In contrast, the BPSN group did not exhibit a large number of macrophage infiltrations around the peritendinous area, including the NF‐κB phosphorylation into the nucleus have decreased and a significantly lower number of CD68^+^ P2Y12^+^ and CD68^+^ CD86^+^ cells, and lower expression of CD31 compared to the PEUU group.

The regulatory mechanism of macrophage activation mediated by the phosphorylation of P2Y12/NF‐κB was verified at the in vivo protein level (Figure 6m). The protein expression of P2Y12 increased or decreased, with a corresponding concomitant increase or decrease in p‐NF‐κB p65 levels. This was consistent with the expectation that CA intervention in fibrous tissue formation resulting from implant‐induced acute‐chronic inflammation or foreign body reaction could be regulated by P2Y12/NF‐κB mediated macrophage polarization. The therapeutic effect of BPSN could be mediated by CD86, P2Y12, p‐p65, CD31, and COLIII protein expression (Figure 6n).

In this study, analysis of human proteomics and adhesion models revealed a marked up‐regulation in the expression of the active P2Y12 molecule, significantly located at the surface of macrophages (Figure S1, Supporting Information). Our further research found that the activation of macrophage‐derived P2Y12 receptor emerged as one of the important factors driving the development of inflammation and peritendinous adhesion. Moreover, based on AI‐based molecular docking simulations, caffeic acid (CA) exhibited a higher affinity for P2Y12 receptors than clopidogrel, as a specific P2Y12 receptor blocker (Figure 1f). To block the activation of P2Y12 receptor and investigate its mechanisms in adhesion formation, BPSN were fabricated using CA molecules as soft segment components. In vitro studies revealed that the controlled release rate of BPSN was stable and long lasting, which in the early stage, the breakage of the ester bond was the dominant factor for slow release, while in the later phase, the ester and carbamate bond both dominate the late stage of BPSN degradation. BPSN inhibited macrophage P2Y12 expression and P2Y12/NF‐κB mediated M1 polarization, closed the ion channels, down‐regulated the secretion of inflammatory factors and disrupted the functional activation of endothelial cells. As shown in our in vivo results, compared with the other two groups, the BPSN treated group exhibited significantly less adhesion formation and positive P2Y12 expression.

## 3 Discussion

Macrophages have been reported as the dominant immune cell type in peritendinous adhesion formation and inflammation related to biomaterial degradation.^[^ ^21^, ^43^, ^44^ ^]^ However, the specific mechanisms and pharmacological interventions targeting the roles of macrophages have not been explicitly addressed. The study indicates that activation of P2Y12 is involved in inflammation, gene deficiency or antagonists of P2Y12 attenuate local inflammation.^[^ ^45^ ^]^ The P2Y12 receptor is an ADP‐activated receptor on the platelet surface.^[^ ^46^ ^]^ The ADP‐dependent pathway activates platelets by inducing the release of intracellular calcium from storage areas. This process also increases extracellular calcium influx and decreases inhibitory signaling, ultimately leading to an elevation in intracellular calcium levels. Elevated intracellular calcium ion concentration promotes phosphorylation of NF‐κB, which in turn activates macrophages to induce inflammation.^[^ ^47^ ^]^ Chen et al. identified by co‐expression of CD68 or F4/80 antigens or macrophage lineage tracing markers found in renal fibrogenesis that up to 82% of P2Y12‐expressing cells were macrophage‐derived.^[^ ^48^ ^]^ In our study, proteomic analysis revealed that P2Y12 is distributed on various cell surfaces, with a higher proportion of macrophages. To further confirm the specificity of P2Y12 expression in macrophages, we performed fluorescent double‐labeling staining for P2Y12 and CD68 on rat Achilles tendon injury sections at 21 days (Figure 1a). The results showed co‐localization of P2Y12 and CD68 expression, indicating that P2Y12 expression originates from macrophages. Furthermore, our molecular docking results indicated a higher affinity between CA and P2Y12 than clopidogrel. The results revealed that the proposed use of CA as an alternative inhibitor to clopidogrel down‐regulated calcium mobilization and blocked P2Y12/NF‐κB mediated aberrant macrophage activation for anti‐adhesion. Thereafter, P2Y12, as found in this study, may be a novel and effective therapeutic target for peritendinous adhesion. Furthermore, the immature neovascular network exacerbates the development of FBR.^[^ ^49^, ^50^ ^]^ This may be related to the inhibition of vascular endothelial growth factor expression by CA.^[^ ^10^ ^]^ Related studies have shown that vascular endothelial cells are activated by inflammatory cytokines (IL‐6), expressing selectins and adhesion factors during the immune response to injured tissue.^[^ ^51^ ^]^ Our results provide a theoretical basis for understanding peritendinous adhesion formation and the potential use of CA in anti‐adhesion treatment.

The high recurrence rate of peritendinous adhesion poses a challenge for clinical treatment. Therefore, the design of a long‐lasting drug‐releasing material to ameliorate inflammation has become necessary. A core‐shell nanofiber membrane, prepared by embedding ibuprofen/Hyaluronic Acid (HA) in silver nanoparticles, prevented early inflammation after tendon surgery by reducing immune cell infiltration.^[^ ^52^ ^]^ However, the abrupt release effect of ibuprofen in this context fails to provide the requisite on‐demand release necessary to mitigate the recurrence of adhesion. In instance, the in vivo ester‐sensitive release of poly‐tioxolone‐loaded Poly‐L‐lactic Acid (PLLA) effectively inhibited adhesion formation.^[^ ^53^ ^]^ Nevertheless, the responsive breakage of single bonds may not be congruent with the later stages of adhesion development, wherein the total loading amount of poly‐tioxolone is established. In this study, we took advantage of the structural characteristics of CA to synthesize a biodegradable copolymer by forming ester and carbamate bonds using CA as a soft segment molecule. This is because CA, as a hydroxycinnamic acid, consists of an aromatic core and conjugated side chains. Consequently, during the initial stages of BPSN biodegradation, the breakage of the ester bonds serves as the predominant mechanism for slow release. In the subsequent phase, both the breakage of ester bonds and carbamate bonds become significant contributors during the late stage of BPSN degradation. This solution addresses the problem of repeated recurrence of peritendinous adhesion that leads to unsatisfactory treatment.

In this study, we discovered that the activation of macrophage P2Y12 was involved in adhesion formation and CA could serve as a blocker for P2Y12, effectively inhibiting the formation of adherent tissues. Furthermore, we introduced a novel anti‐inflammatory method by creating a biodegradable PEUU‐CA‐derived BPSN capable of presenting a persistent CA niche locally. This mediated CA‐release kinetics effectively addressed the clinical obstacle of peritendinous adhesion.

## 4 Experimental Section

### Synthesis of PEUU‐CA

PEUU‐CA was synthesized through a two‐step solution polymerization:^[^ ^54^ ^]^ PCL diol and HDI were mixed and reacted at 80 °C for 2 h, followed by the addition of CA for chain extension at 40 °C for at least 18 h until the residual ‐NCO groups were completely consumed. The molar ratio of PCL diol, CA, HDI, and BDA was 0.85:0.15:2:1.

### Preparation and Characterization of Nanofibers

Electrospinning solution for preparing PEUU nanofibers and PEUU‐CA nanofibers (bioactive patches with sustainable niches, BPSN) according to the following protocol: 1g of PEUU and 1g of PEUU‐CA were dissolved separately in 10 mL of HFIP, resulting in mixtures with a 10% concentration, stirred until clear. Using electrospinning equipment, random nanofiber mats and tubes were produced. The solutions were loaded into a 10 mL syringe with a 20‐gauge stainless‐steel needle, connected to a high‐pressure source with an output voltage of 10.3 kV. The nanofibers produced through electrospinning were deposited onto either a rotating stainless‐steel bar or a flat aluminum foil board positioned 14 cm away from the capillary. This resulted in the formation of nanofiber patches designated as PEUU and BPSN, respectively. Subsequently, the prepared nanofibers underwent a 48‐hour vacuuming process in a desiccator to eliminate any remaining HFIP residues. The surface morphology of the nanofibers was observed under a scanning electron microscopy (S‐4800, Hitachi, Tokyo, Japan) and the nanofiber diameter was measured. The surface wettability of PEUU and BPSN were tested at room temperature using a water contact angle analyzer (DSA25S; Data Physics Corporation, San Jose, CA, USA).

### In Vitro Drug Release

PEUU and BPSN were randomly cut into 6 blocks of ≈20 mg for each sample. They were soaked in 3 mL of phosphate‐buffered saline (PBS) (containing lipase 0.05 mg mL^−1^) and placed in a horizontal shaking water bath (Taichang Medical Apparatus Co., Jiangsu, China) at 37 °C for co‐incubation. Extracts were aspirated at each time point (0, 1, 3, 5, 7, 10, 14, 21, 28, 42, and 56 days). Based on the measured standard absorption curve of caffeic acid (R^2^ = 0.9984), the concentration of caffeic acid sustained released from BPSN was detected by UV spectrophotometry (UV‐2550, Shimadzu, Japan). And calculate the percentage release of caffeic acid under each time point based on the loading of the caffeic acid in the BPSN.

### Patches Weightlessness

To further evaluate the patches degradation properties of BPSN, we calculated the weight of the patches in order to measure drug release as described above. We trimmed BPSN into discs of the same size to weigh. As mentioned above, BPSN was immersed in 3 mL of PBS and sealed with parafilm. This was done by aspirating the BPSN extract, then spreading the patches as far as possible, placing it in a desiccant bottle, and drying it in an oven at 70°C. After cooling, the patches were weighed airtight and the percentage weight loss of BPSN was calculated and analyzed separately for each time point (0, 1, 3, 5, 7, 10, 14, 21, 28, 42, and 56 days). The percentage weight loss was calculated as follows:

(1) $$\mathrm{Remaining}\;\mathrm{loss}\;\left(\%\right)=\left(1-\frac{W-W^{\prime }}{W}\right)*100\%$$

W and W´ were the dry weight of the BPSN before and after biodegradation, respectively.

### Artificial Intelligence‐Based Protein Structure Modeling and Molecular Docking

Alpha folding is an artificial intelligence prediction tool that can predict protein structures with high accuracy based on their amino acid sequences, matching wet‐lab experiments.^[^ ^55^ ^]^ Although RCSB Protein Data Bank has not published the protein crystal structure of rat P2Y12, we performed in silico modeling to construct the structure of P2Y12. The amino acid sequence of P2Y12 was downloaded from the UniProt‐KB database and generated the predicted protein structure by AlphaFold v2.0. The stereochemical quality of the predicted models was checked using the Local Distance Difference Test (LDDT) scoring of the AlphaFold database, ERRAT, and Preservation v6.0 items.

Predicting the orientation of small molecules to form stable complexes with macromolecular targets by molecular docking simulations. Briefly, clopidogrel (P2Y12 inhibitor) was downloaded from the Pubchem database.^[^ ^56^ ^]^ Implementation of AutoDockTools and PyMol (DeLano Science, CA, USA) pre‐processed the input files, including hydrogenation of water and ligands and deletion. Molecular docking between CA or clopidogrel and the P2Y12 binding pocket was performed by Autodockvina using default parameters. The geometry of predicted CA binding interactions with clopidogrel or P2Y12 were visualized and scored for docking affinity between small molecules and protein targets. The optimal conformation of CA or clopidogrel and P2Y12 were achieved by PyMol method.

### Cell Adhesion Assay

Macrophage was grown in degradation solution in 24‐well plates. Based on the results of in vitro drug release, the degradation solution of PEUU and BPSN was selected for 6 w to culture cells. After 24 h, the cells were washed and fixed, and stained with Phalloidin‐iFluor 488 according to the manufacturer's instructions. Morphologic differences in cytoskeletal arrangement observed under the fluorescence microscopy (DMi8, Leica microsystems Co. Ltd).

### Cell Culture and Migration and Tube Formation Assay

The RAW264.7 macrophages and HUVECs were cultured in DMEM containing 10% FBS incubator at 37 °C with 5% CO_2_. Macrophages were grown in 24‐well plates at 60% confluency in different concentrations of caffeic acid or degradation solution and incubated for 24 h at 37°C. For induction of M1 polarization, the cells were pretreated with LPS (1 µg mL^−1^) for 4 h. To assess the crosstalk between macrophages and endothelial cells, the supernatant was collected. HUVECs were cultivated in Transwell chambers (5×10^5^ cells per well), and incubated with supernatant. 24 h later, the HUVECs were fixed for 10 min. After washing, the cells were stained with crystal violet for 15 min, and the number of migrated cells was observed under the microscopy.

Tube formation assay was performed as described previously with modification.^[^ ^57^ ^]^ HUVECs were cultured in 6‐well plates at 1×10^5^ cells and treated with macrophage supernatant for 24 h when 60% fusion was reached Matrigel (Corning, 356230) was added in 96‐well plates (all operations were performed on ice) and placed in a 37 °C constant temperature incubator for 40 min. After the gel be solid, the above cells were cultured in the gel for 4 hours. Tube formation was observed under the microscopy.

### Cell Proliferation Assay

RAW267.4 cells were planted in 96‐well plates with 1×10^4^ cells per well and incubated overnight at 37°C. Upon treatment, different concentrations of caffeic acid were added to macrophages to co‐culture for 24h. HUVECs were seeded in 96‐well plates at 60% confluency. After the cells adhered to the wall, the cell culture medium was changed into supernatant of treated macrophages. Cell proliferation was detected by CCK‐8 assay kit according to the instructions for utilization, and the absorption wavelengths of the samples at 450 nm were determined spectrophotometrically. All the sample supernatants were divided into 3 parts as technical 3 replicates, and the statistical results of each group were biological 3 replicates.

### Immunofluorescence Assay

Macrophages were incubated in 24‐well plates with different concentration gradients of caffeic acid for 24 h, flushed thrice with PBS solution, fixed for 20 min, 0.5% tritonX‐100 membrane‐breaking for 15 min, and 5% goat serum closure for 30 min, and then incubated with primary antibodies overnight at 4°C. Cells were washed with PBS and co‐incubated with fluorescent secondary antibody for 2 h and DAPI for 15 min at room temperature. Cells were cultured in 96‐well plates and processed as described above. Add Calcium Probe Detection Reagent and incubate at 37 °C for 40 min and DAPI for 5 min, followed by observation under the fluorescence microscopy, and cells were imaged with 6 randomly selected fields of view per well. Antibody information is as follows:

P2Y12, Abclonal, A20864, 1:50

CD86, Abclonal, A16805, 1:100

iNOS, Abclonal, A3774, 1:200

Alexa Fluor 488, Proteintech, SA00013‐2, 1:200

Alexa Fluor 594, Proteintech, SA00013‐4, 1:200

### Real‐Time PCR

Total RNA from the cultured cells was extracted by using TRIzol total RNA reagent. Complementary DNA (cDNA) was synthesized by using HiScript III RT SuperMix (Vazyme, R323‐01) according to the manufacturer's protocol. For the RT‐qPCR analysis, SYBR qPCR Master Mix (Vazyme, Q711‐02) was utilized in a thermal cycler, alongside two unique primer sets designed for each target gene. Normalization with GAPDH was performed to quantify 2^−ΔΔ Ct^ and the relative gene expression was calculated by the 2^−ΔΔCT^ method.

### Elisa

Macrophage supernatants were collected to detect TNF‐α, IL‐6, and VEGF expression by elisa test. Briefly, 100 µL of the diluted sample was added to the reaction wells coated with the appropriate antibody and incubated for 90 min at 37°C. After washing 3 to 5 times, added 100 µL of the biotinylated secondary antibody and incubated for 1 h at 37°C. After washing, the avidin‐biotin‐peroxidase complex was added and incubated at 37 °C for 30 min. Then the samples were incubated with TMB chromogenic reagent at 37 °C in dark for 30 min. Added 100 µL termination solution to stop reaction. The protein concentration was calculated based on the reference standard curve which the absorbance was measured at 450 nm.

### Achilles Tendon Injury Model

All animal study protocols were approved by the Institutional Review Board of Shanghai Jiao Tong University (SYXK (Hu) 2016‐0020). Male Sprague‐Dawley rats at 6 weeks were selected and anesthetized with pentobarbital intravenously. Hind limbs were sterilized and incised to expose and dissect the Achilles tendon. Repair was performed using a modified Kessler technique and 6‐0 nonabsorbable sutures. The damaged Achilles tendon was wrapped with PEUU or BPSN, and the control group was left untreated.

### Gross Evaluation

After postoperative euthanasia of 4 w and 8 w rats, the repaired Achilles tendon was exposed again and the degree of peritendinous adhesion was assessed according to the quantitative scoring system on a scale of 1 to 5 (Figure S13, Supporting Information).^[^ ^58^ ^]^

### Biomechanical Evaluation

Biomechanical testing was performed at 4 w and 8 w postoperatively. The Achilles tendon and surrounding tissue at the surgical site was taken ≈1 to 2 cm and fixed to the tensile tester. The proximal and distal ends of the Achilles tendon were clamped (Figure S11, Supporting Information) and pulled proximally at 30 mm min^−1^ until the Achilles tendon could no longer be stretched or ruptured, and the maximum load was recorded to evaluate the biological strength of the repaired tendon. Mechanical‐tension diagrams for each sample were counted by a rheometer.

### Histological Evaluation

The hind limbs of rats were taken, and fixed in paraformaldehyde for 48 h and decalcified with 10% ethylenediaminetetraacetic acid for 8 w. Tissues were paraffin‐embedded and sectioned into 5 µm thin slices. Sections were stained with hematoxylin‐eosin, Masson's trichrome stain and immunohistochemical stain of collagen III. The sections were observed under a light microscope (Leica DM6000). The adhesion histological scoring system was shown in Figure S14 (Supporting Information).^[^ ^58^ ^]^

### RNA‐Seq

The peritendinous adherent tissues wrapped by PEUU and BPSN were taken, ground in liquid nitrogen and added with Biozol Reagent, and incubated at room temperature for 2 min for RNA extraction. Take 1µL Qubit4.0 instrument (invitrogen, USA) for quantification, and detect the RNA quality according to the quantification results. The construction of RNA library included mRNA isolation and fragmentation, double‐stranded cDNA synthesis, addition of A junction and PCR enrichment, and the RNA library was quantified by Qubit (invitrogen, USA). After the above work, the samples were subjected to novaseq 6000 (illumnia, USA) on the machine.

### Western Blot

Western bolt analysis was performed as previously described.^[^ ^59^ ^]^ Briefly, peritendinous adhesion tissues were lysed in RIPA lysis buffer for extracting protein. And the protein (20µg) was separated by 10% SDS‐PAGE at 100 V for 70 min and transferred onto PVDF membranes at 300 mA for 45 min. The membranes were blocked by 5% skim milk for 2 h and incubated with primary antibodies overnight at 4 °C and then with secondary antibodies for 2 h at room temperature. The following antibodies were used: β‐actin (ab8227) (1:1000; Abcam, UK), CD31 (A19014), CD86 (A16805), P2Y12 (A20864) (1:2000; ABclonal), p‐P65 (93H1) and COLIII (98908) (1:1000; Cell Signaling Technology, USA). Proteins were visualized, and the relative protein expression were normalized to β‐actin band using Image‐J software.

### Statistical Analysis

All experiments were repeated more than 3 times and expressed as mean ± SD. GraphPad Prism 8.0 and SPSS 10.0 software (IBM Corp, Armonk, New York, USA) were used for statistical analysis. Student's *t* test was used between two groups, one‐way analysis of variance and Fisher's exact test were used between groups, and P < 0.05 indicated that the difference was statistically significant.

## Conflict of Interest

The authors declare no conflict of interest.

## Author Contributions

Z.T., S.W., and J.L. contributed equally to this work. Z.T., S.W., T.Z., J.J., S.L., and X.M. performed conceptualization. Z.T., J.L., and T.Z. performed the methodology. Z.T., S.W., and J.L. performed the investigation. Z.T., S.W., T.Z., and S.L. performed visualization. T.Z., S.L., and J.J. performed supervision. Z.T., J.L., S.L. performed writing—original draft. T.Z., J.J., S.L., and X.M. performed writing—review & editing.

## Supporting information



Supporting Information

## Data Availability

The data that support the findings of this study are available from the corresponding author upon reasonable request.
