# Supplementary material for: Sustainable Immunomodulatory via Macrophage P2Y12 Inhibition Mediated Bioactive Patche for Peritendinous Antiadhesion
Source: Adv Sci (Weinh). 2024 Dec 4;12(4):2409128. doi: 10.1002/advs.202409128 (PMC11775537; doi:10.1002/advs.202409128)
Supplement: Supplementary file 1 — Supporting Information [file ADVS-12-2409128-s001.docx]

Supporting Information for

Sustainable Immunomodulatory via Macrophage P2Y12 inhibition Mediated Bioactive Patche for Peritendinous Antiadhesion

Zaijin Tao, Shuo Wang, Jingwen Liu, Tonghe Zhu^*^, Jia Jiang^*^, Shen Liu^*^, Xin Ma^*^

Correspondence to: [maxin@sjtu.edu.cn](mailto:maxin@sjtu.edu.cn)


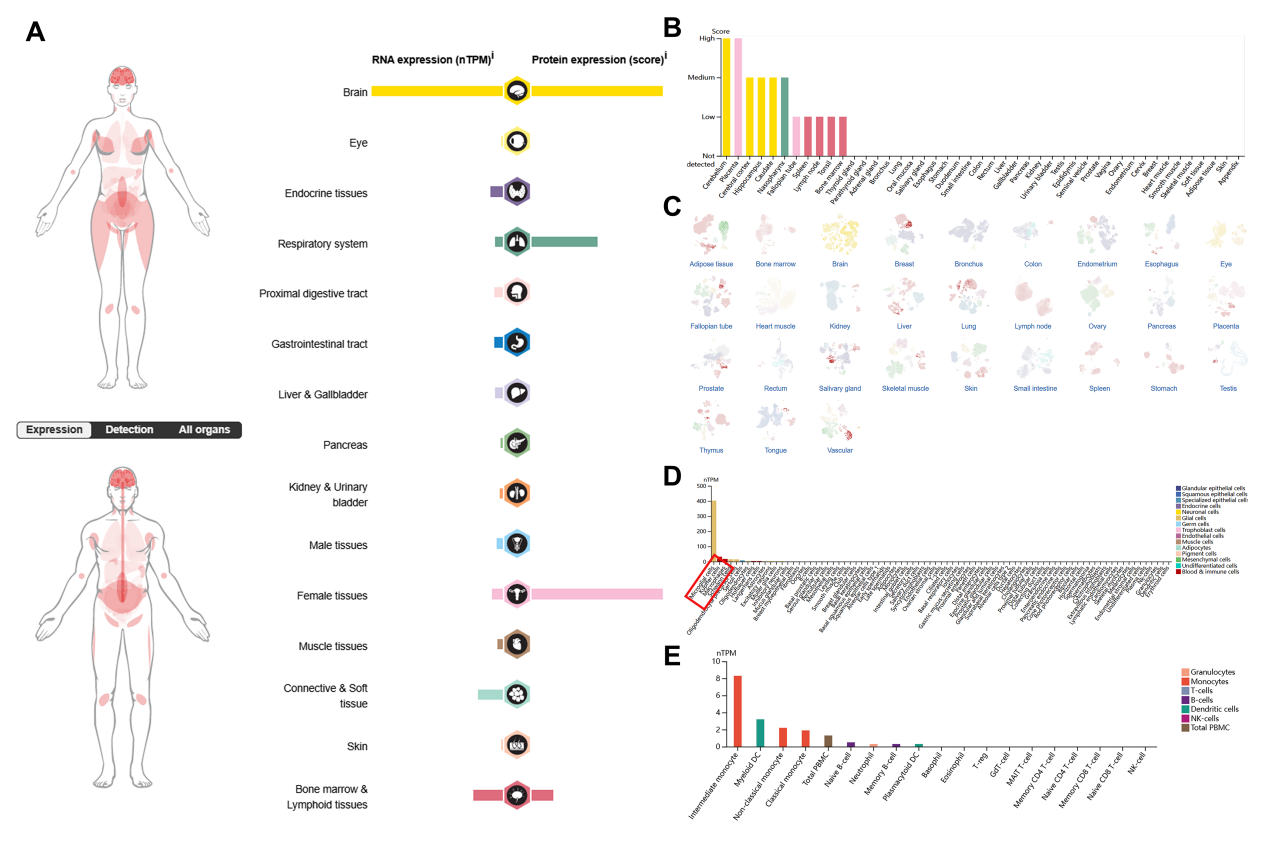
Fig. S1. (A-C) Distribution of P2Y12 in human tissues and organs. (D-E) Histogram of P2Y12 distribution in immune cells. (Microglial cells, Kupffer cells, Macrophages, Schwann cells are markered with a red box.)


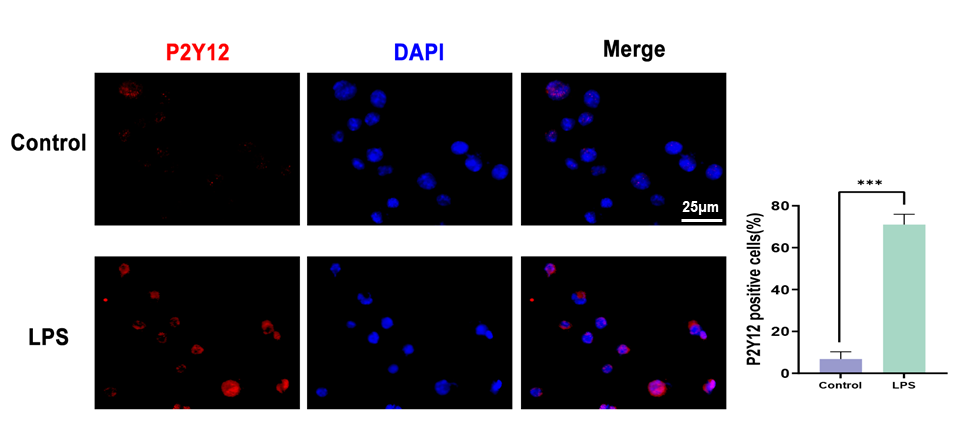
Fig. S2. Validation of macrophage surface P2Y12 expression at the cellular level. (Data are representatives of independent experiments and all data are given as means ± SD; n=5 per group; ns: non-significant, P > 0.05; *P < 0.05; **P < 0.01; ***P < 0.001; ****P < 0.0001).


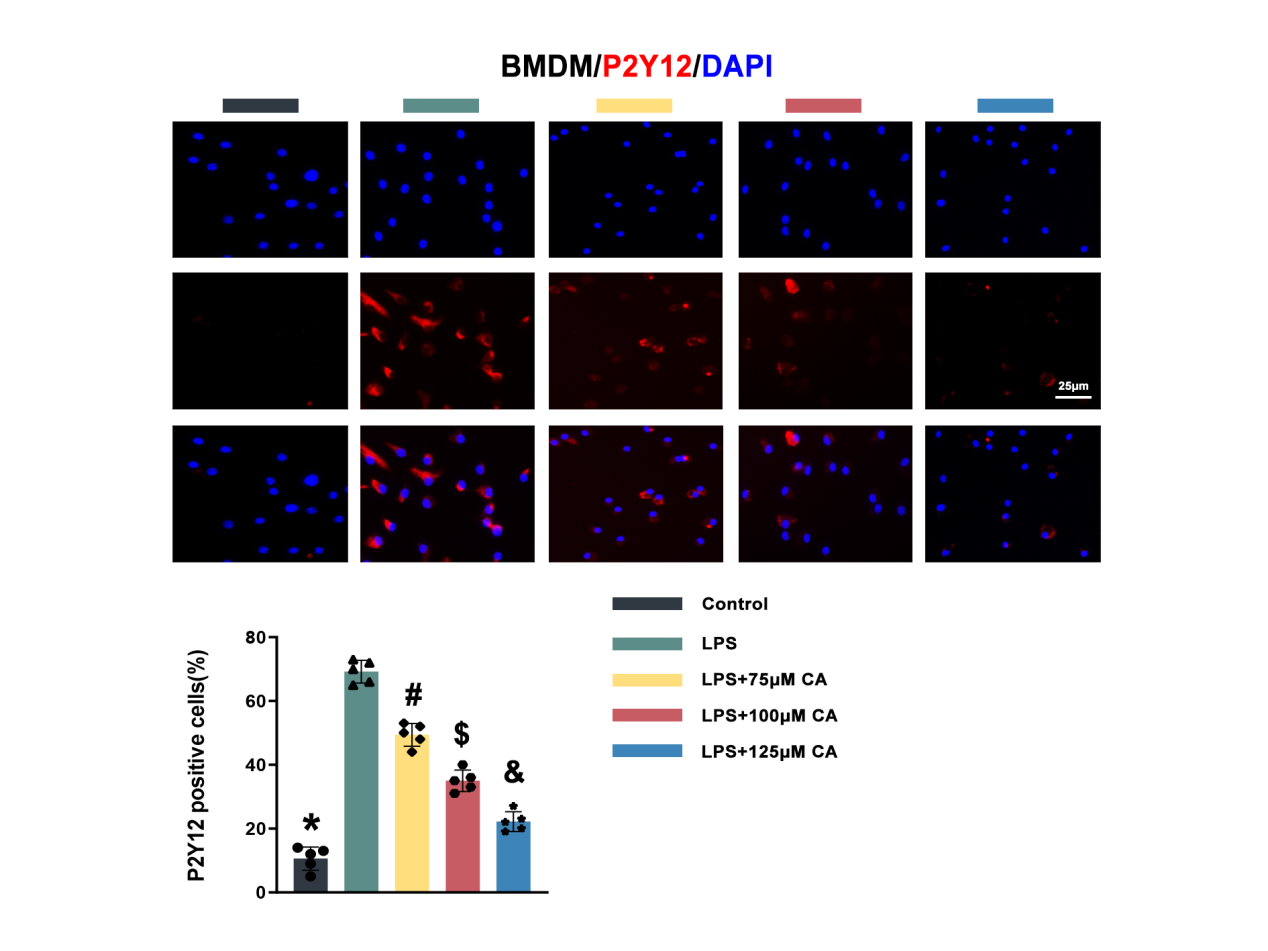


Fig. S3. Inhibition of P2Y12 receptor expression on the surface of BMDM by CA and statistical quantitative analysis. (Data are representatives of independent experiments and all data are given as means ± SD; n=5 per group; *, #, $, & p <0.05 compared to the LPS group).


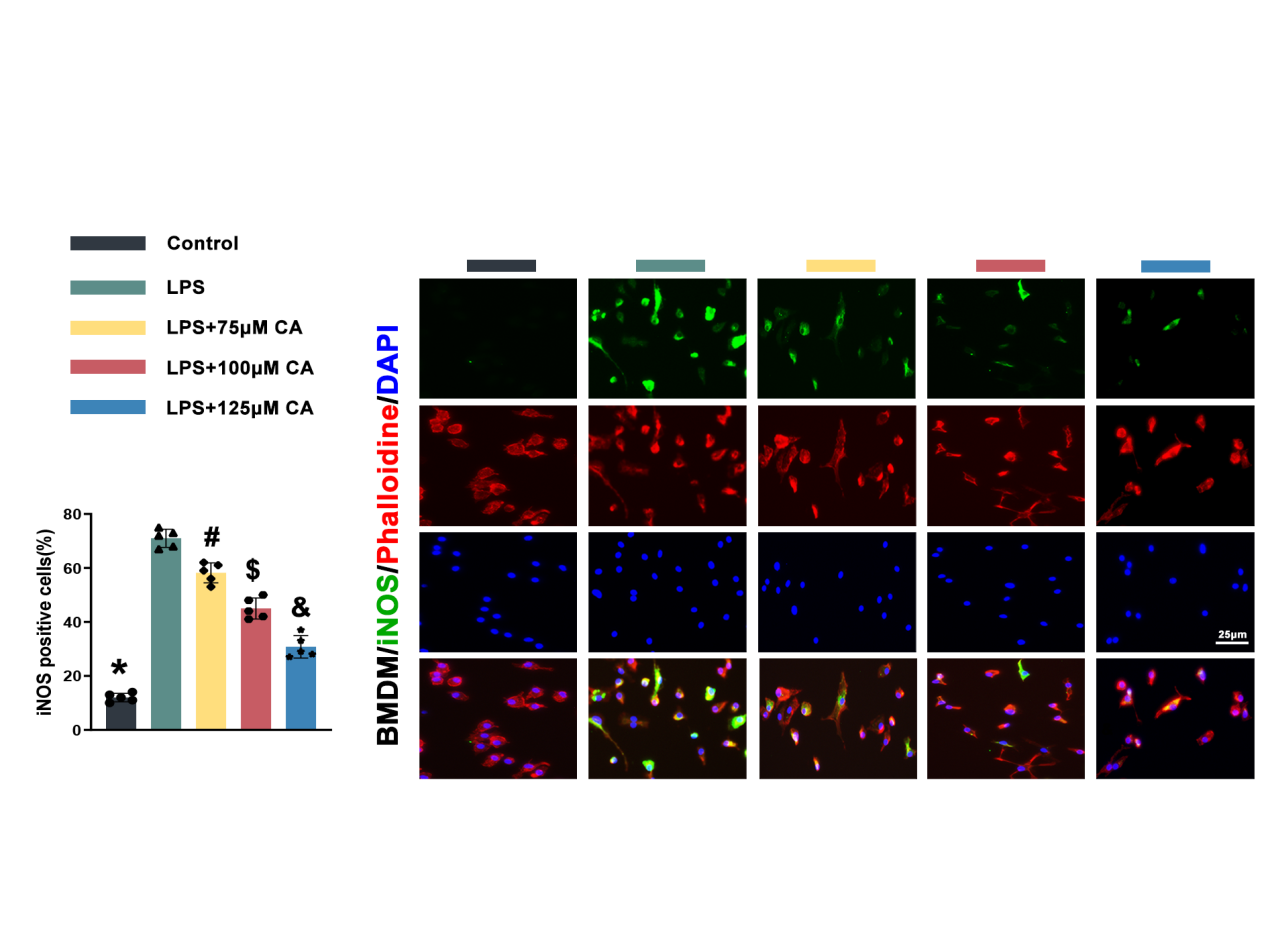
Fig. S4. iNOS/Phalloidine fluorescence double staining was used to analyze the anti-inflammatory performance of CA at different concentrations, and the positive cellular iNOS expression rate statistics. (Data are representatives of independent experiments and all data are given as means ± SD; n=5 per group; *, #, $, & p <0.05 compared to the LPS group).


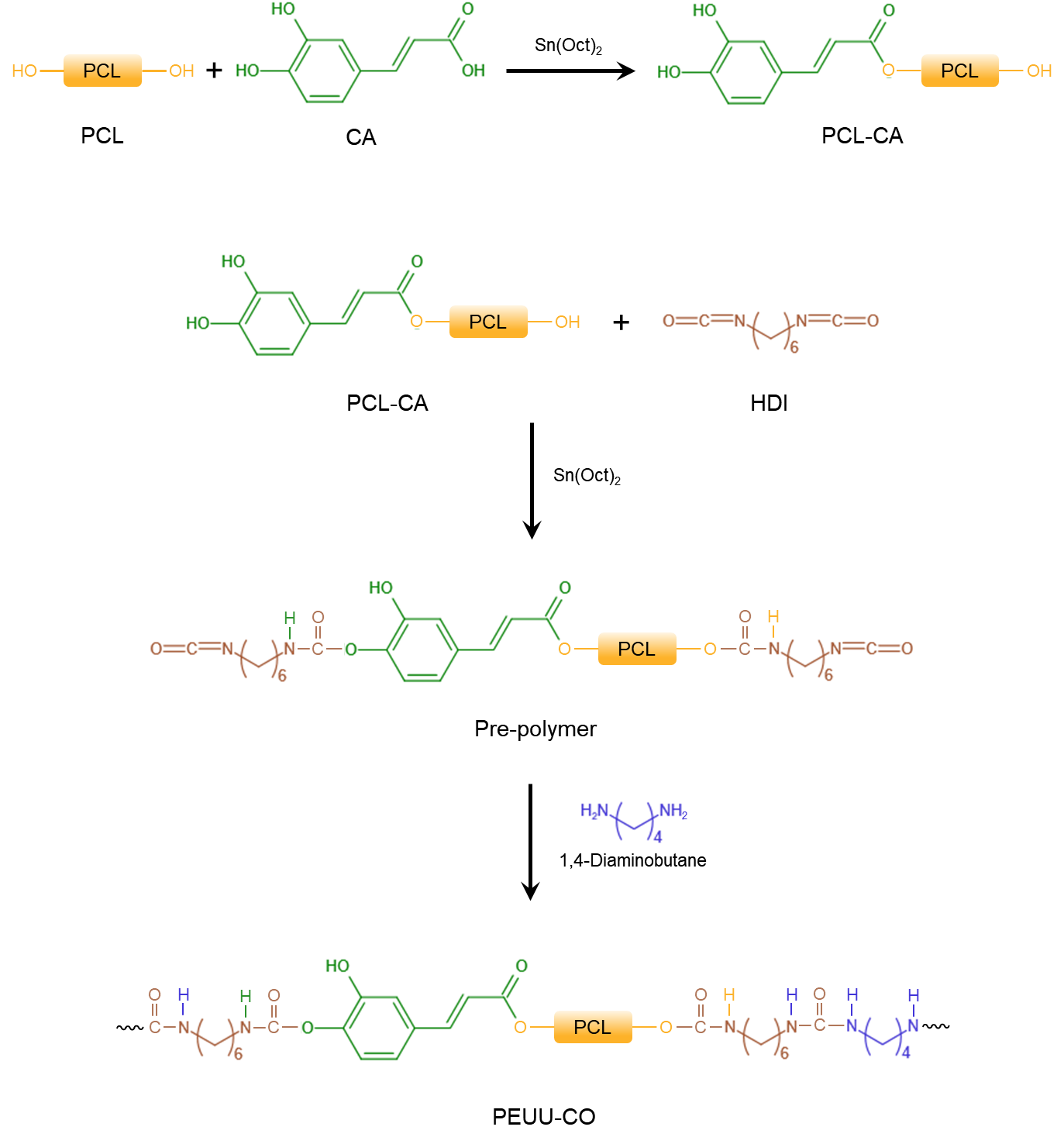


Fig. S5. Schematic diagram of BPSN synthesis.


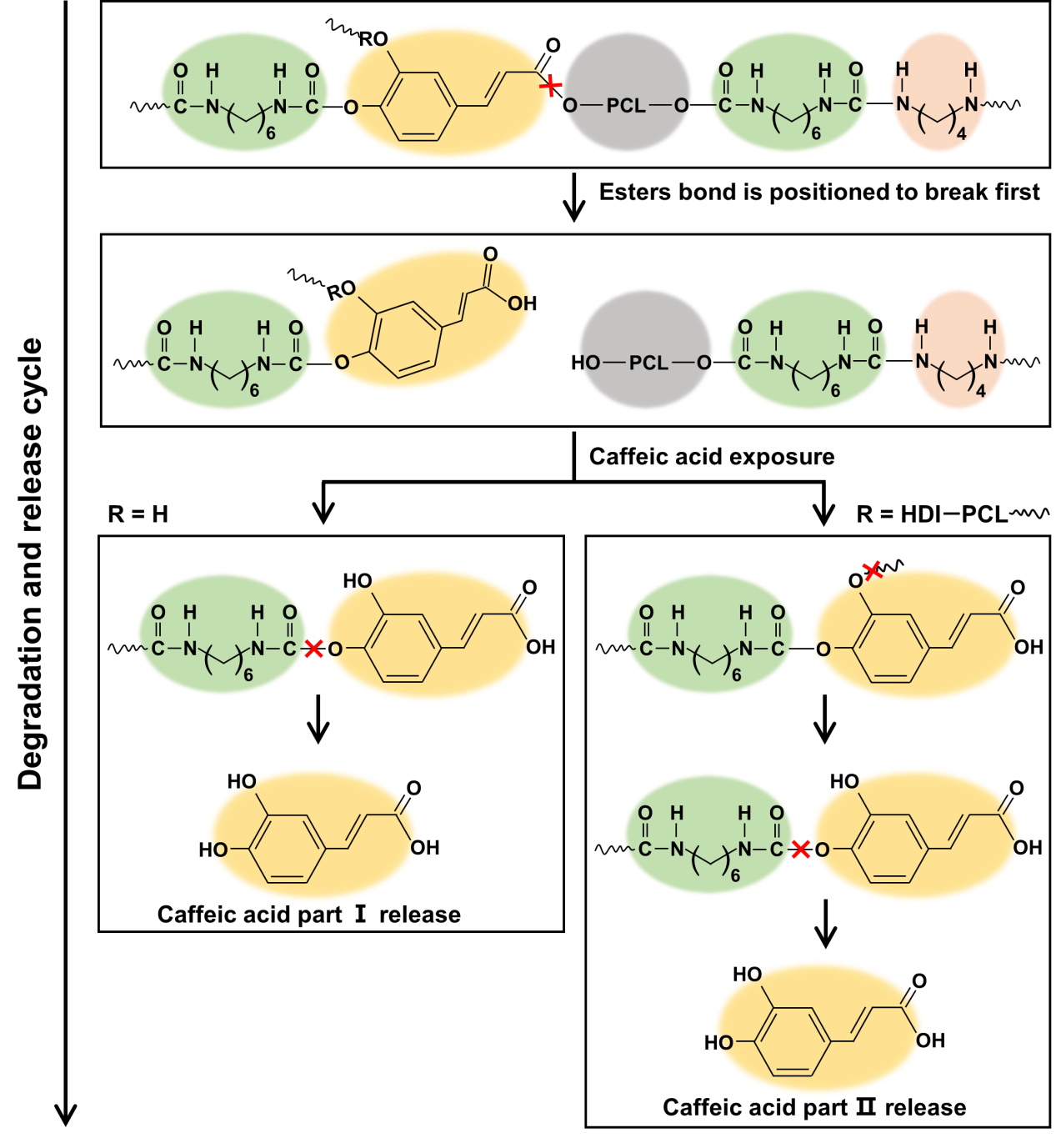
Fig. S6. Schematic diagram of BPSN degradation.


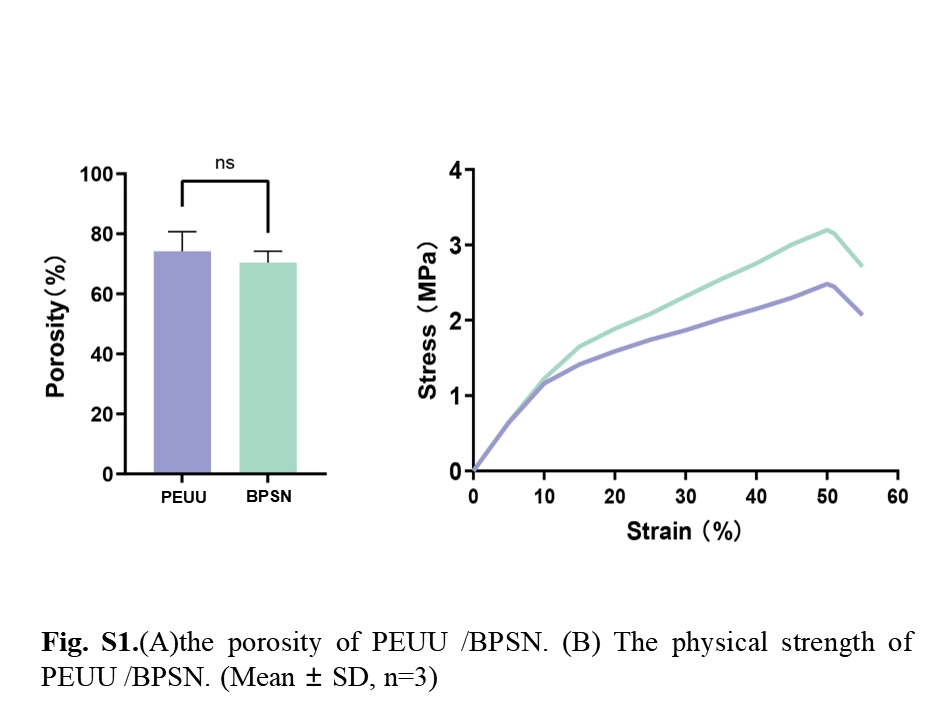
Fig. S7. The porosity of PEUU /BPSN. (B) The physical strength of PEUU /BPSN. (Mean ± SD, n=3; ns: non-significant.)


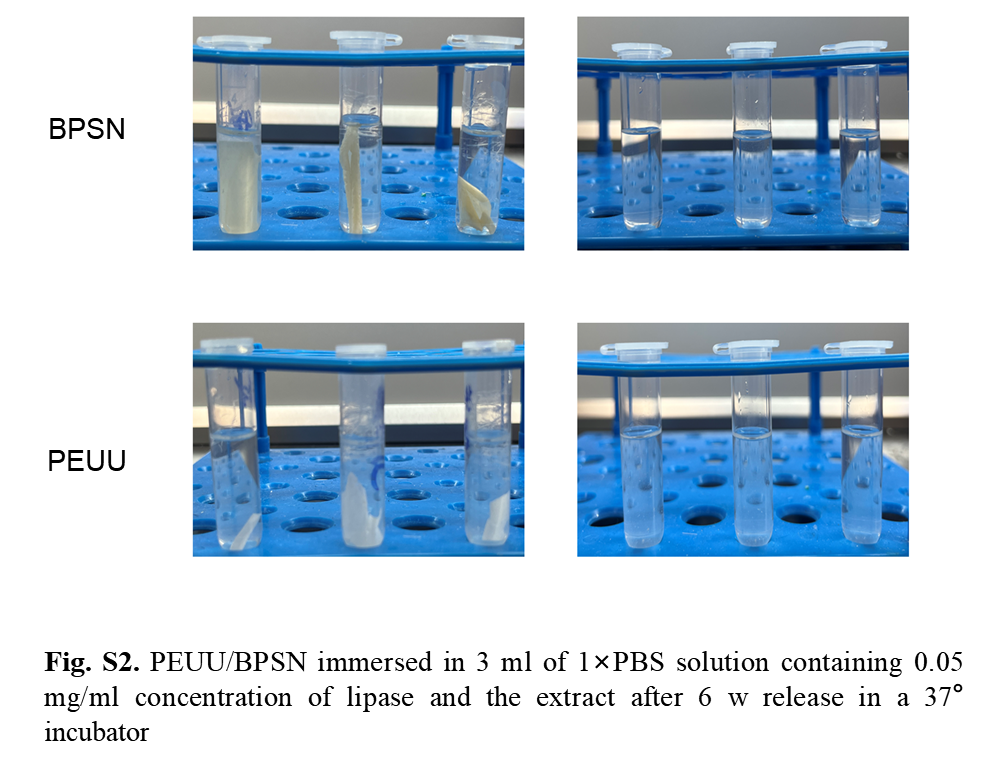
Fig. S8. PEUU/BPSN immersed in 3 mL of 1 × PBS solution containing 0.05 mg/mL concentration of lipase and the extract after 6 w release in a 37 ℃ incubator.


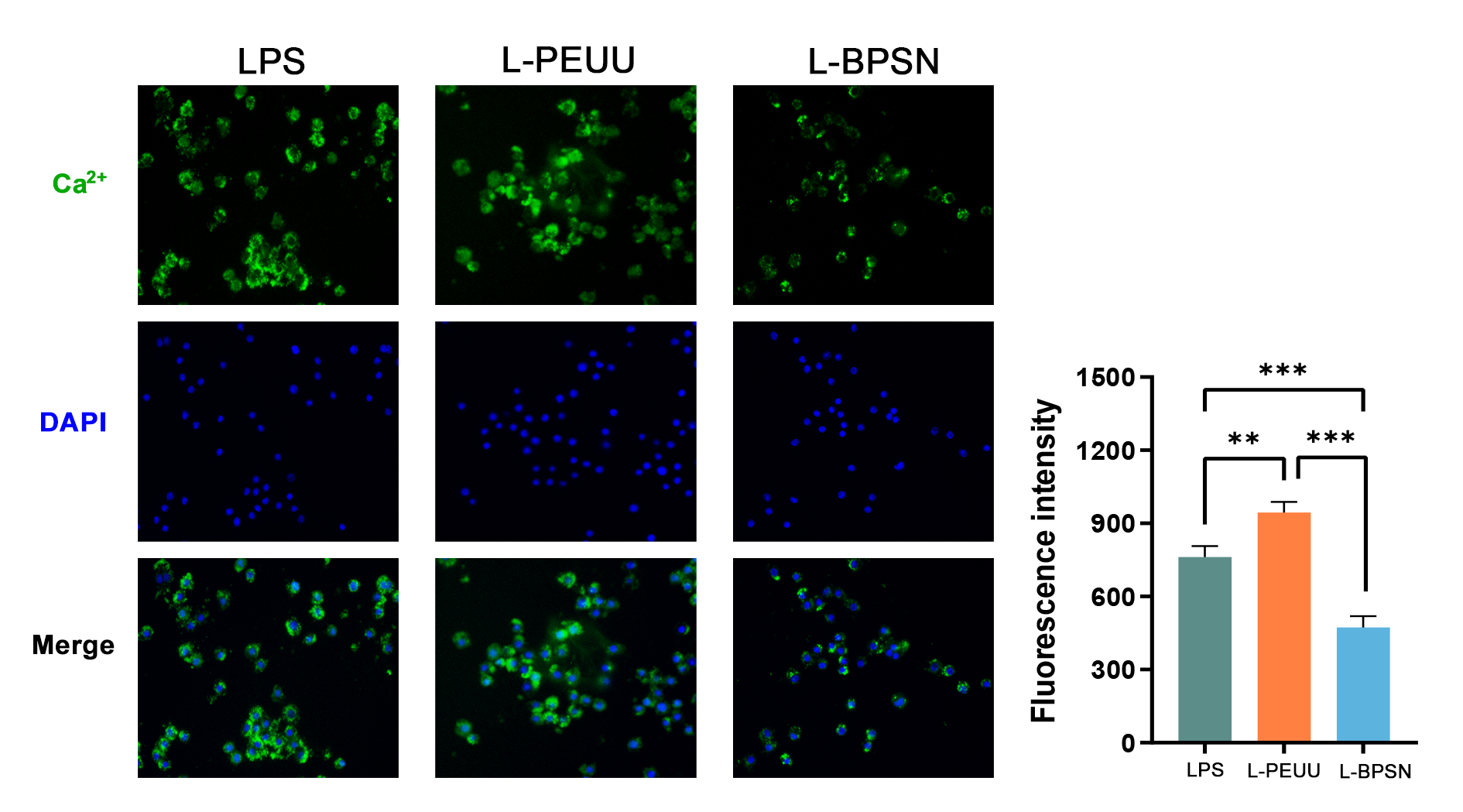


Fig. S9. PEUU/BPSN extracts were co-cultured with macrophages and calcium ions were detected in each group using fluorescent labeling with a calcium ion probe. Green fluorescence represents Ca^2+^ and blue DAPI represents nuclei. Intracellular and extracellular fluorescence intensity of macrophages in each group. (Data are representatives of independent experiments and all data are given as means ± SD; n=3 per group; ns: non-significant, P > 0.05; *P < 0.05; **P < 0.01; ***P < 0.001; ****P < 0.0001).


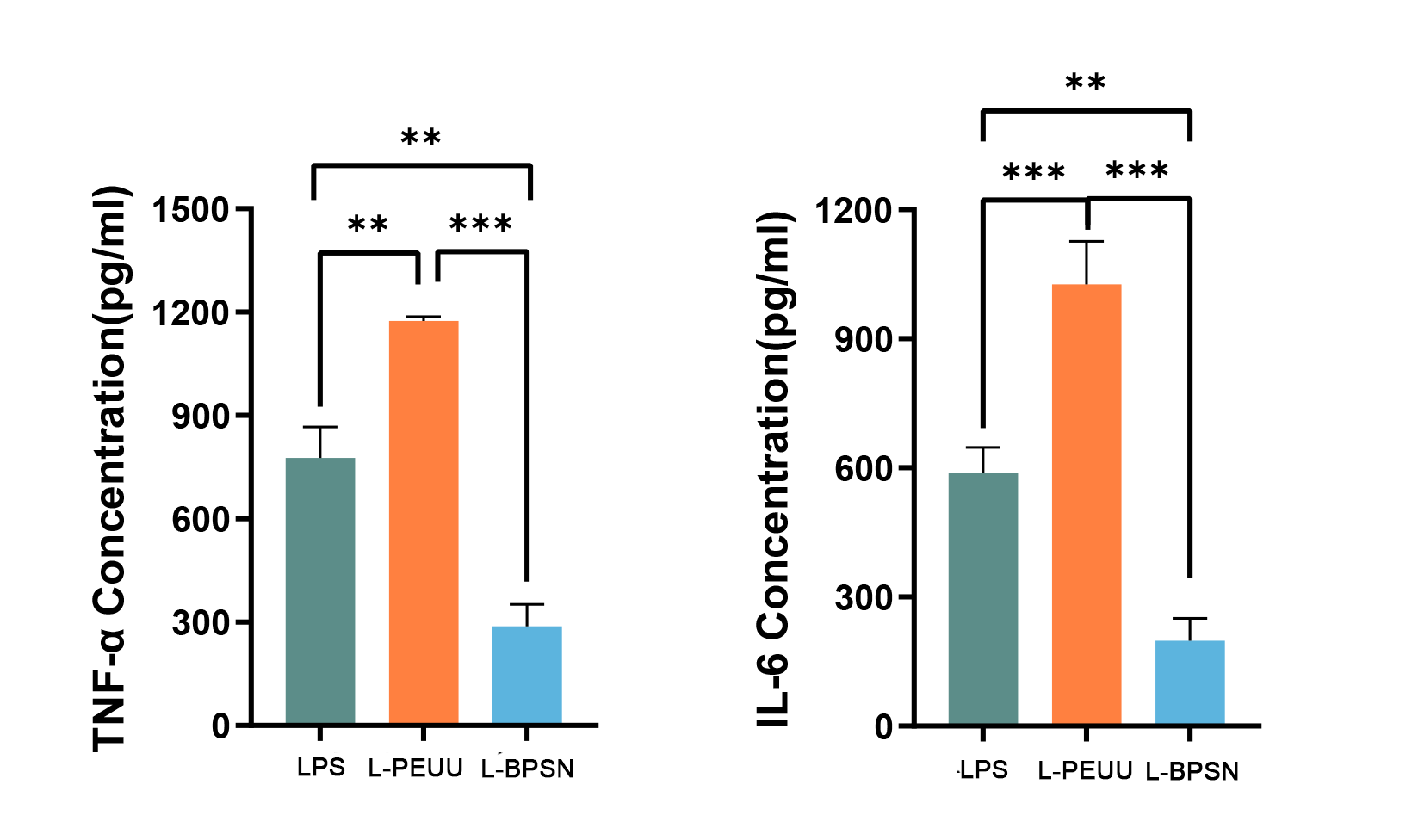


Fig. S10. PEUU/BPSN extracts were co-cultured with macrophages, and the concentration of TNF-α and IL-6 in supernatant evaluated by Elisa. (Data are representatives of independent experiments and all data are given as means ± SD; n=3 per group; ns: non-significant, P > 0.05; *P < 0.05; **P < 0.01; ***P < 0.001; ****P < 0.0001).


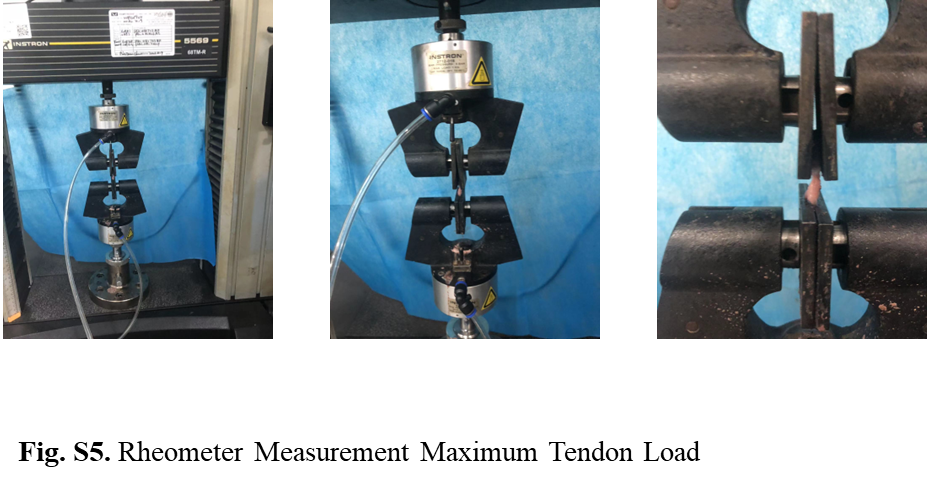
Fig. S11. Rheometer Measurement Maximum Tendon Load.


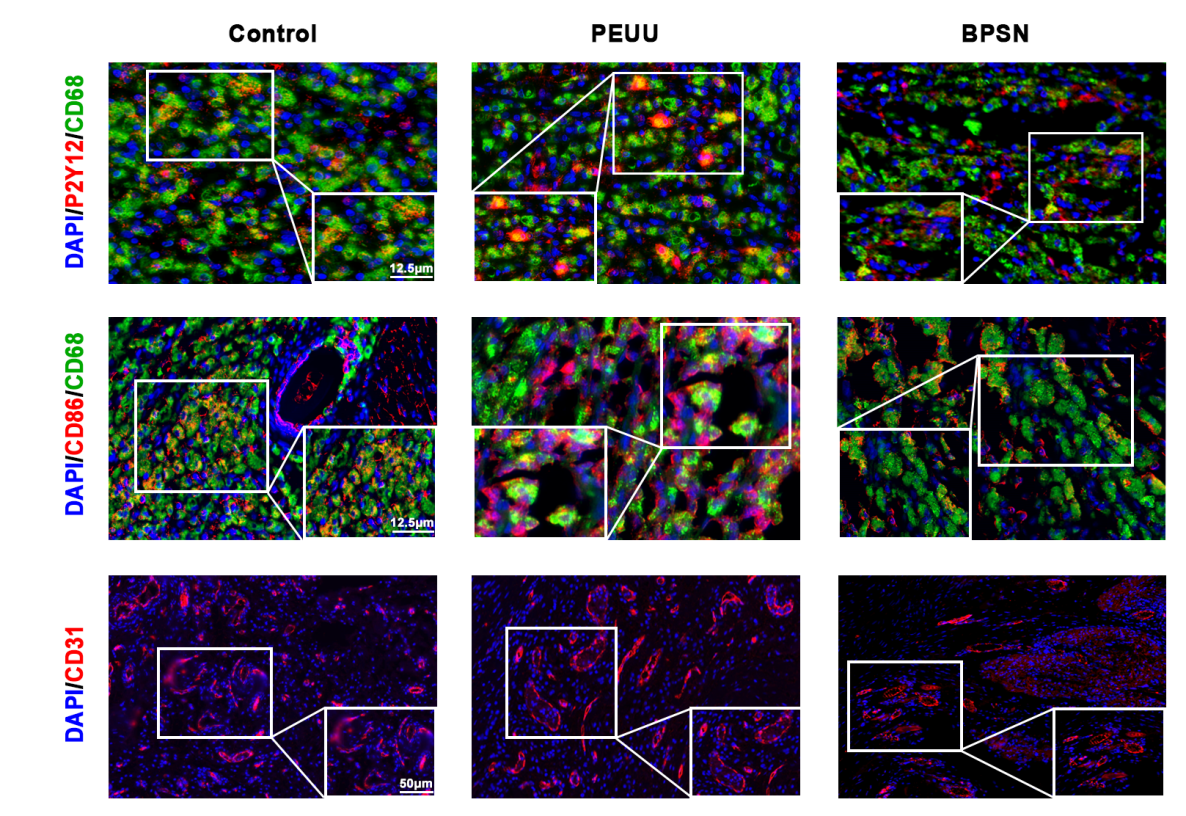


Fig. S12. Immunofluorescence staining results of CD68/P2Y12, CD68/CD86 and CD31 at 4 w postoperatively.


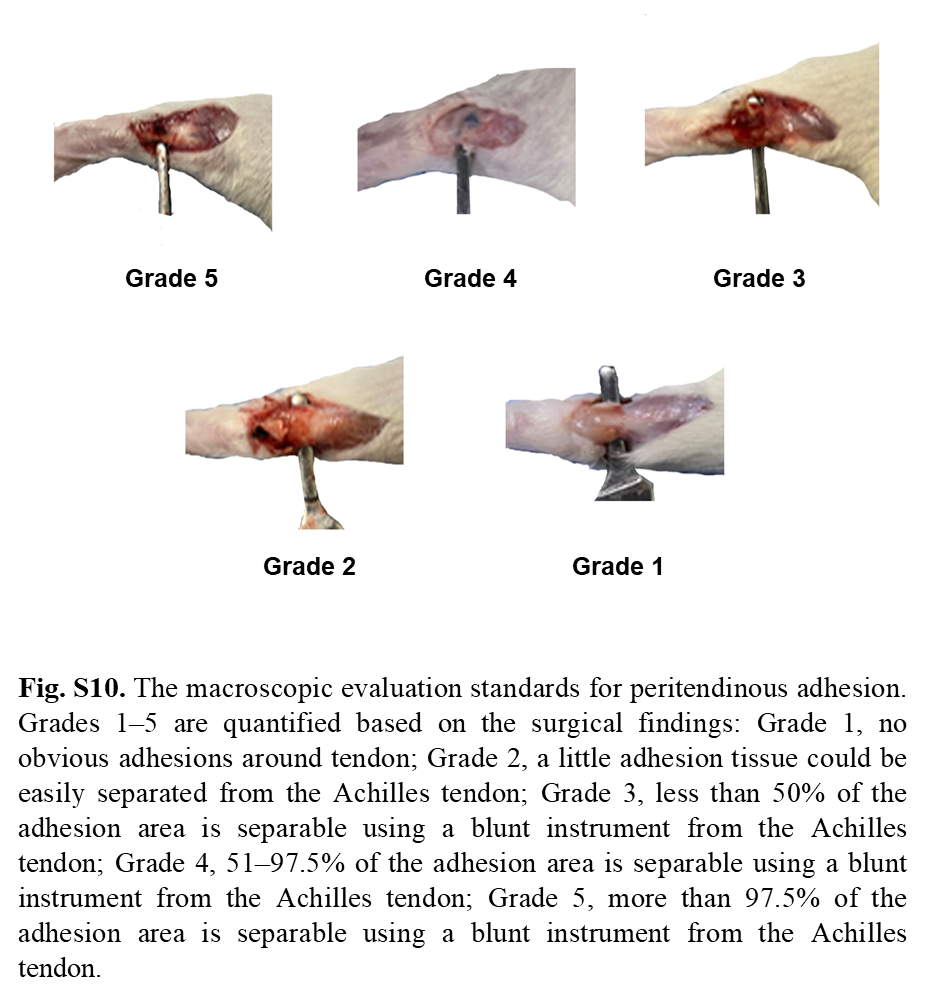
Fig. S13. The macroscopic evaluation standards for peritendinous adhesion. Grades 1–5 are quantified based on the surgical findings: Grade 1, no obvious adhesion around tendon; Grade 2, a little adhesion tissue could be easily separated from the Achilles tendon; Grade 3, less than 50% of the adhesion area is separable using a blunt instrument from the Achilles tendon; Grade 4, 51–97.5% of the adhesion area is separable using a blunt instrument from the Achilles tendon; Grade 5, more than 97.5% of the adhesion area is separable using a blunt instrument from the Achilles tendon.


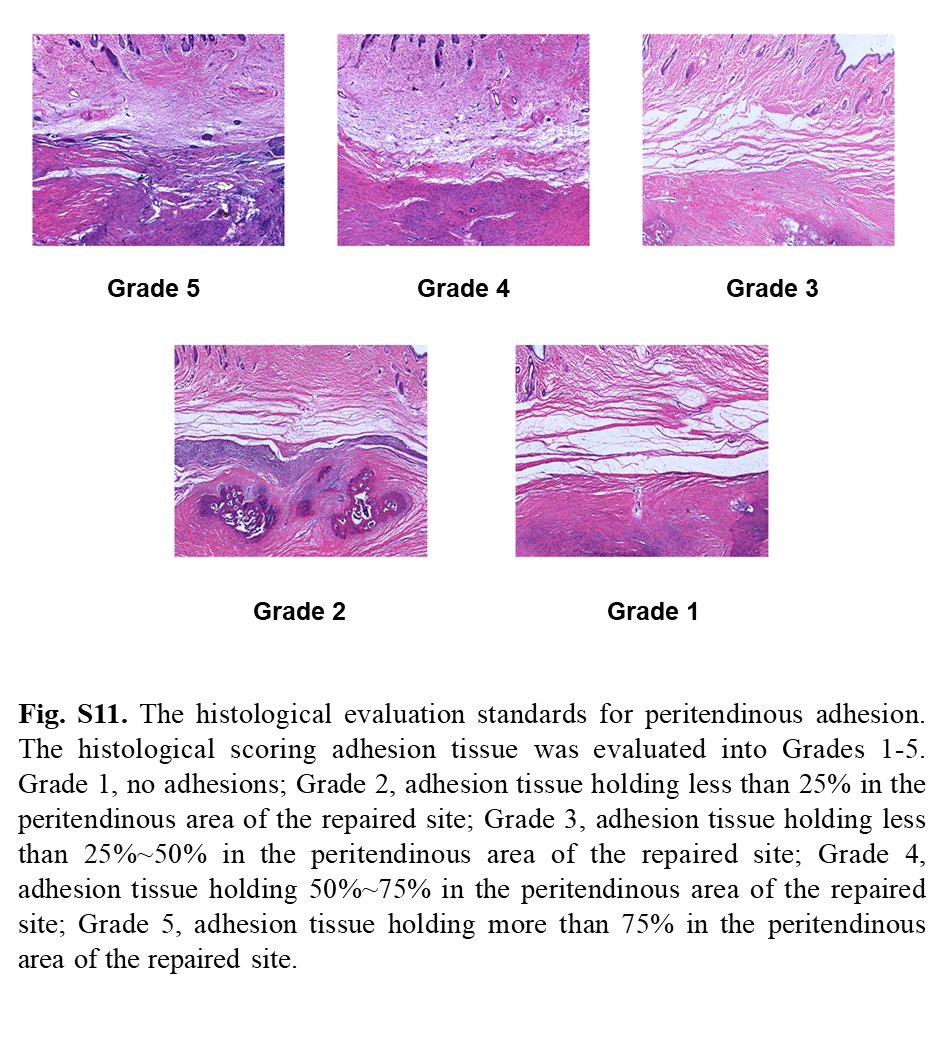
Fig. S14. The histological evaluation standards for peritendinous adhesion. The histological scoring adhesion tissue was evaluated into Grades 1-5. Grade 1, no adhesion; Grade 2, adhesion tissue holding less than 25% in the peritendinous area of the repaired site; Grade 3, adhesion tissue holding less than 25%~50% in the peritendinous area of the repaired site; Grade 4, adhesion tissue holding 50%~75% in the peritendinous area of the repaired site; Grade 5, adhesion tissue holding more than 75% in the peritendinous area of the repaired site.


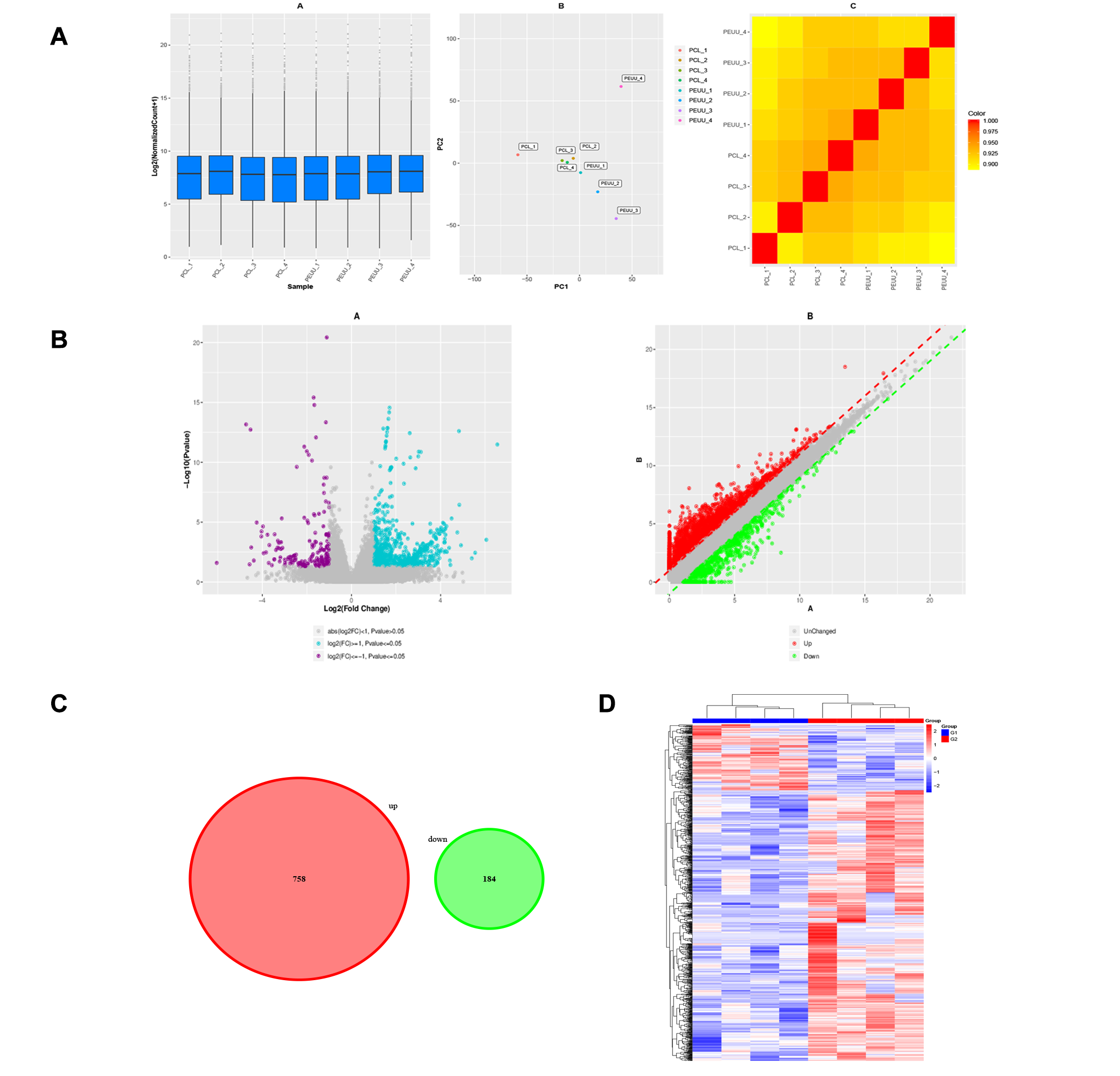
Fig. S15. Comparative analysis of overall gene expression levels. (A) Known gene expression abundance Boxplot (Boxplot), PCA plot (PCA) and sample correlation coefficient matrix heatmap (Correlation). (B) Volcano and scatter plots between two groups of samples. (C) Differential Gene Screening Venn Diagram. (D) For the differentially expressed genes screened between sample groups, a two-way hierarchical clustering of genes and samples was used and shown in a heat map.


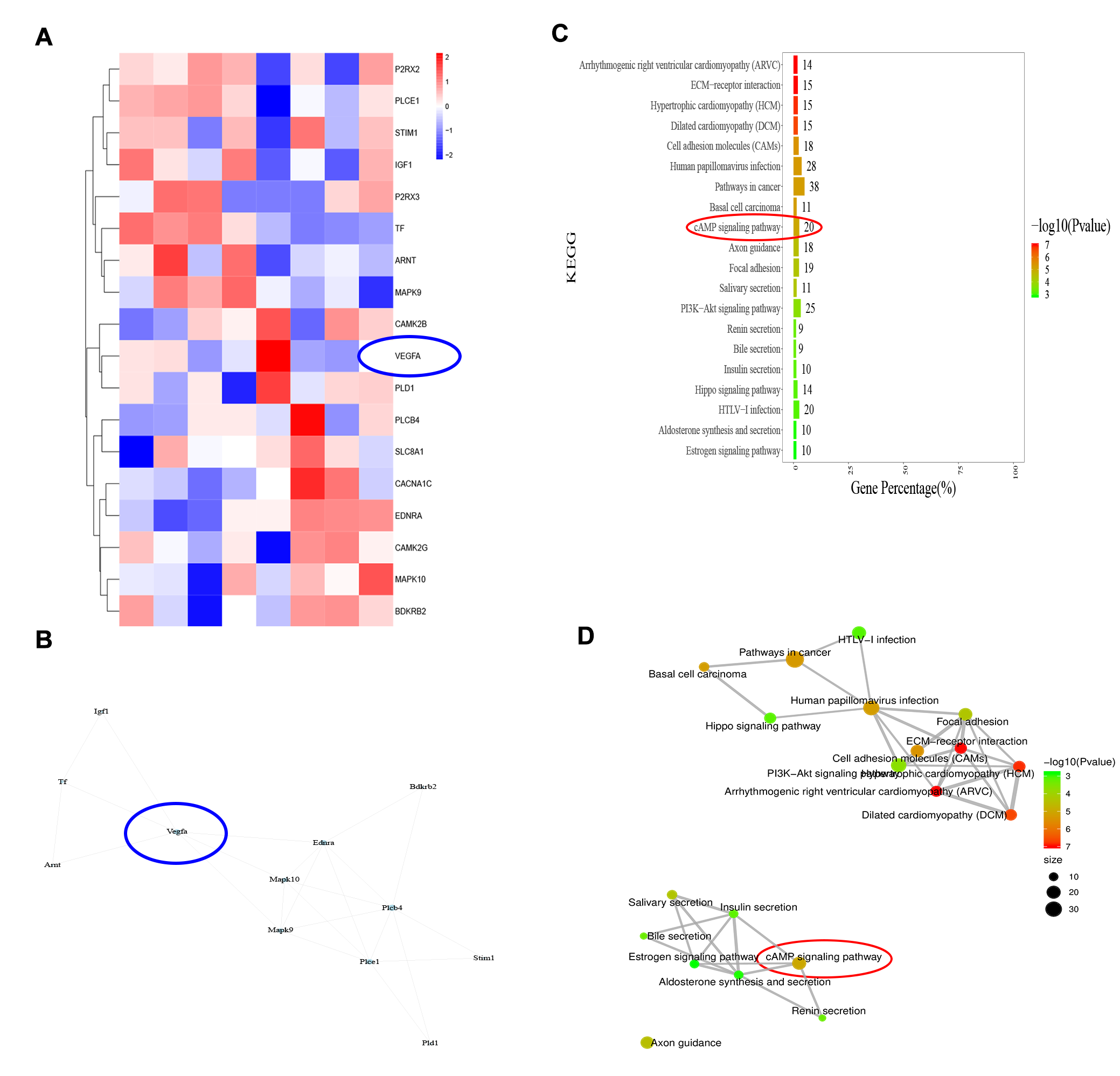


Fig. S16. Target gene set KEGG metabolic pathway analysis. (A-B) Heatmap and protein interaction network diagram of genes down-regulated in the KEGG metabolic pathway related to the comparison of PEUU and BPSN groups (VEGF). (B) Scatter plot of KEGG significantly enriched in up-regulated genes and (C) network plot of KEGG vs. KEGG (cAMP signaling pathway).


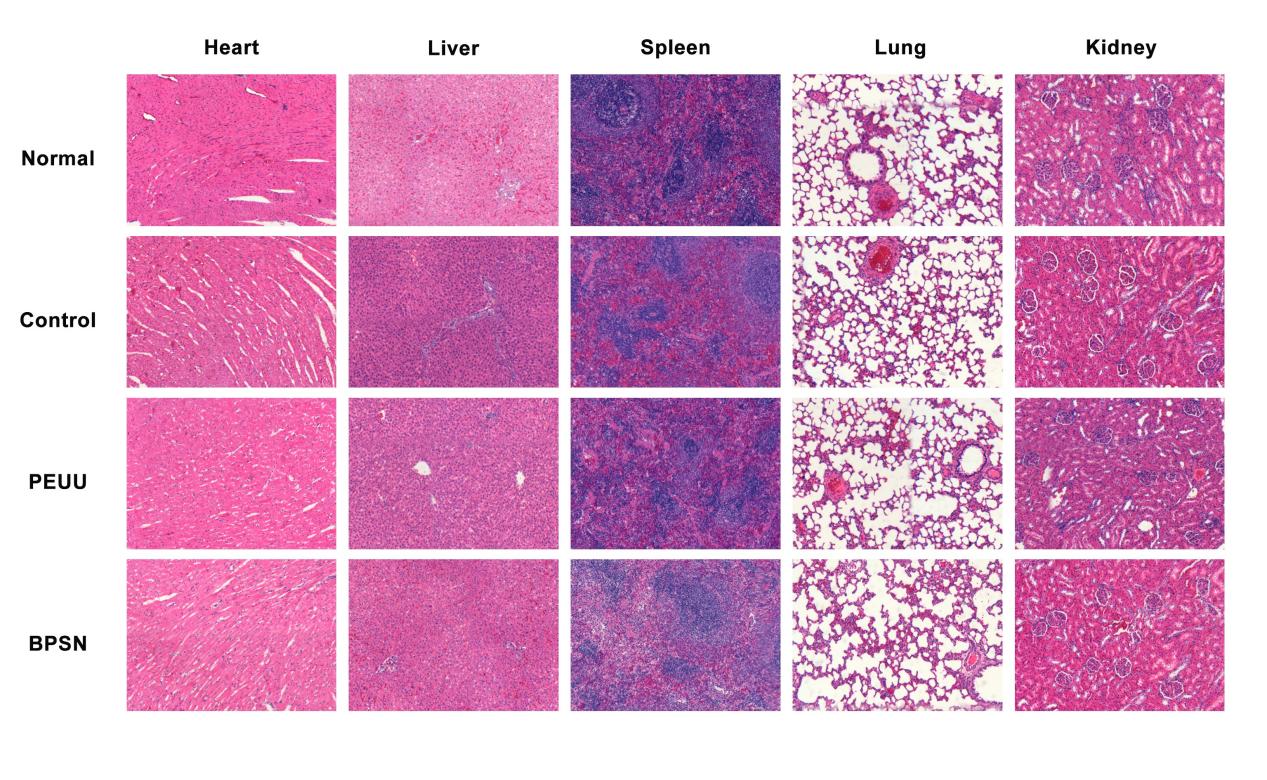


Fig. S17. H&E staining images of heart, liver, spleen, lung, and kidney sections from rat on the 8th weeks.
